# Adolescent social housing protects against adult emotional and cognitive deficits and alters the PFC and NAc transcriptome in male and female C57BL/6J mice

**DOI:** 10.3389/fnins.2023.1287584

**Published:** 2023-12-07

**Authors:** Jyoti Lodha, Emily R. Brocato, McKenzie Nash, Madison M. Marcus, A. Chris Pais, Alex B. Pais, Michael F. Miles, Jennifer Theresa Wolstenholme

**Affiliations:** ^1^Pharmacology and Toxicology Department, Virginia Commonwealth University, Richmond, VA, United States; ^2^VCU Alcohol Research Center, Virginia Commonwealth University, Richmond, VA, United States

**Keywords:** adolescence, alcohol, stress, social interaction, transcriptomics, sex differences

## Abstract

**Introduction:**

Adolescence is a critical period in cognitive and emotional development, characterized by high levels of social interaction and increases in risk-taking behavior including binge drinking. Adolescent exposure to social stress and binge ethanol have individually been associated with the development of social, emotional, and cognitive deficits, as well as increased risk for alcohol use disorder. Disruption of cortical development by early life social stress and/or binge drinking may partly underlie these enduring emotional, cognitive, and behavioral effects. The study goal is to implement a novel neighbor housing environment to identify the effects of adolescent neighbor housing and/or binge ethanol drinking on (1) a battery of emotional and cognitive tasks (2) adult ethanol drinking behavior, and (3) the nucleus accumbens and prefrontal cortex transcriptome.

**Methods:**

Adolescent male and female C57BL/6J mice were single or neighbor housed with or without access to intermittent ethanol. One cohort underwent behavioral testing during adulthood to determine social preference, expression of anxiety-like behavior, cognitive performance, and patterns of ethanol intake. The second cohort was sacrificed in late adolescence and brain tissue was used for transcriptomics analysis.

**Results:**

As adults, single housed mice displayed decreased social interaction, deficits in the novel object recognition task, and increased anxiety-like behavior, relative to neighbor-housed mice. There was no effect of housing condition on adolescent or adult ethanol consumption. Adolescent ethanol exposure did not alter adult ethanol intake. Transcriptomics analysis revealed that adolescent housing condition and ethanol exposure resulted in differential expression of genes related to synaptic plasticity in the nucleus accumbens and genes related to methylation, the extracellular matrix and inflammation in the prefrontal cortex.

**Discussion:**

The behavioral results indicate that social interaction during adolescence via the neighbor housing model may protect against emotional, social, and cognitive deficits. In addition, the transcriptomics results suggest that these behavioral alterations may be mediated in part by dysregulation of transcription in the frontal cortex or the nucleus accumbens.

## 1 Introduction

Adolescence is characterized by high levels of playful social interactions, cognitive development, and increased risk-taking behaviors including binge drinking (Spear, 2000). In most species, adolescents spend more time interacting with peers than during any other developmental period (Spear, 2000; van Kerkhof et al., 2013). Adolescents are less sensitive to the social impairing, motor disrupting, aversive, and sedative effects of higher doses of ethanol compared to adults. Conversely, adolescents tend to be more sensitive to the rewarding effects of ethanol (Spear, 2018), long term memory deficits, and delayed frontal cortex development when compared to adults (Lodha and Brocato, 2022). Together, this may enable adolescents to engage in the binge drinking behavior commonly seen within this age group (Spear, 2014; Spear, 2018). Early life stress also tends to increase drug-seeking behavior in underage populations. In a longitudinal study, a portion of the population that drinks in isolation during the adolescent/young adult period is at high risk of developing an AUD by age 35; this is especially true for females (Creswell et al., 2022). In college students, increased feelings of loneliness and a tendency to drink as a means of coping with stress was associated with heavy drinking (Gonzalez et al., 2009; Gonzalez and Skewes, 2013). Furthermore, the risk of developing an alcohol use disorder (AUD) in adulthood increases the younger one first engages in alcohol consumption (Grant and Dawson, 1997; Spear, 2000).

Adolescent exposure to social isolation or binge ethanol causes lasting structural and molecular changes in brain development, as well as behavioral alterations that last into adulthood. For example, social isolation or binge ethanol can reduce myelin content in the frontal cortex (De Bellis et al., 2005; Medina et al., 2008; Makinodan et al., 2012; Vargas et al., 2014; Montesinos et al., 2015; Vetreno et al., 2016; Wolstenholme et al., 2017; Pfefferbaum et al., 2018; Hinton et al., 2019; Tavares et al., 2019; El Marroun et al., 2021), disrupt dopamine signaling in the nucleus accumbens (NAc) (Yorgason et al., 2013, 2016; Karkhanis et al., 2014; Zhang et al., 2021) and prefrontal cortex (PFC) (Trantham-Davidson et al., 2014, 2017), and disrupt proper functioning of the hypothalamic–pituitary–adrenal (HPA) axis (Butler et al., 2014; Varlinskaya et al., 2015, 2017; Hinton et al., 2019). Behaviorally, these early life experiences can lead to memory deficits (Bianchi et al., 2006; Pascual et al., 2007; Koike et al., 2009; McLean et al., 2010; Vargas et al., 2014; Montesinos et al., 2015; Vetreno and Crews, 2015; Vetreno et al., 2016; Marco et al., 2017; Wolstenholme et al., 2017; Pais et al., 2019; Pascual et al., 2021; Bent et al., 2022), changes in social behavior (Varlinskaya and Spear, 2002, 2004; Kercmar et al., 2011; Makinodan et al., 2012; Varlinskaya et al., 2014; Liu et al., 2015; Lander et al., 2017; Varlinskaya et al., 2017; Medendorp et al., 2018; Liu et al., 2019; Rivera-Irizarry et al., 2020; Zhang et al., 2021), anxiety-like behavior (Weiss et al., 2004; Võikar et al., 2005; Koike et al., 2009; McCool and Chappell, 2009; Chappell et al., 2013; Yorgason et al., 2013; Gass et al., 2014; Karkhanis et al., 2014; Lopez and Laber, 2015; Pandey et al., 2015; Skelly et al., 2015; Montesinos et al., 2016; Sakharkar et al., 2016; Vetreno et al., 2016; Lander et al., 2017; Lee et al., 2017, 2018; Szumlinski et al., 2019; Rivera-Irizarry et al., 2020), and increased drinking in adulthood (Deehan et al., 2007; McCool and Chappell, 2009; Moore et al., 2010; Strong et al., 2010; Lopez et al., 2011; Metten et al., 2011; Sanna et al., 2011; Chappell et al., 2013; Butler et al., 2014; Lesscher et al., 2015, 2021; Skelly et al., 2015; Montesinos et al., 2016; Carrara-Nascimento PF et al., 2017; Lee et al., 2017; Younis et al., 2019; Wolstenholme et al., 2020).

Given the overlapping effects of ethanol use and social isolation (Lodha and Brocato, 2022), the widely used protocol of single housing mice for drinking studies can result in interpretive complications. The presence or absence of social peers becomes a particularly important consideration when modeling adolescent drinking behavior. In the current study, we implement a neighbor housing model which allows mice to have partial social contact with two neighboring mice and may alleviate aspects of the social stress associated with single housing (Pais et al., 2019). The current studies begin to determine whether neighbor housing can ameliorate changes in the development of the prefrontal cortex and nucleus accumbens from social isolation and thus model typical adolescent development while allowing for individual ethanol drinking. Social and basal anxiety-like behaviors were reduced, while cognitive deficits in the novel object recognition task were rescued by neighbor housing. Surprisingly, single housing in adolescence did not alter ethanol intake or preference in adolescent males or females. Early access to ethanol in adolescence did not increase adult intake in neighbor or single housed mice. Our bioinformatics analysis in the NAc strongly suggests alterations in synaptic plasticity due to housing and ethanol drinking. In the PFC, we saw changes to genes involved in methylation, the extracellular matrix, and inflammation due to housing and ethanol drinking. In our qPCR follow-up study, we saw a cluster of immediate early genes were differentially altered by ethanol drinking depending on their housing condition, suggesting that social experience can modulate activity-dependent responses in the PFC following ethanol drinking. Together, these data show that single housing during the critical adolescent period increases social and anxiety-like behaviors and leads to cognitive deficits in adulthood, some of which may be mediated by alterations in the PFC and NAc transcriptome. Neighbor housing alleviates some of these changes perhaps by providing a more enriched social environment. However, drinking behavior was not modulated by adolescent social isolation or neighbor housing.

## 2 Materials and methods

### 2.1 Animals and housing

**Figure 1:**
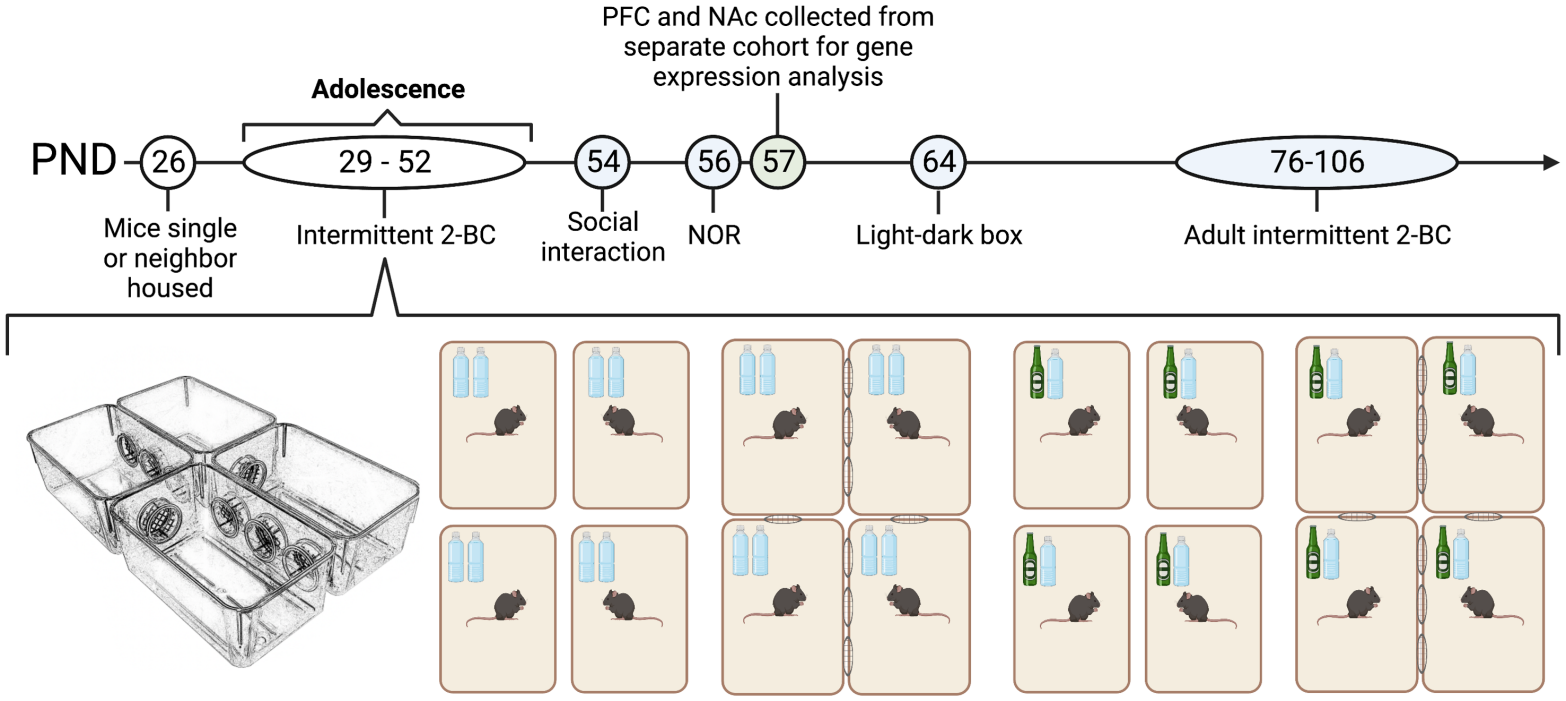
Experimental design male and female C57BL/6J mice were housed in same-sex groups in one of two conditions from post-natal day 29 (PND 29) until the end of the experiment: neighbor housed (4/complex) or single housed (1/cage). Single and neighbor-housed animals were given intermittent access to two bottles of water or a two-bottle choice between ethanol and water. Blue PNDs on the timeline indicate experiments run for the first cohort of animals, while green PNDs indicate experiments run for the second cohort of animals. In cohort one, behavioral testing for male (*n* = 36) and female (*n* = 36) animals began on PND 54. The assays tested were social interaction, novel object recognition (NOR), and anxiety-like behavior in the light–dark box. Adult ethanol intake and preference was also measured where all animals were given access to a two-bottle choice between ethanol and water from PND 76–106. In cohort two, male (*n* = 16) and female (*n* = 16) were single and neighbor housed and given access to intermittent ethanol or water concurrently with cohort one. On PND 57, PFC, and NAc tissue was collected for microarray and qPCR analysis. Male and female cohorts were run separately.

Male and female C57BL/6J mice from Jackson Laboratory (Bar Harbor, ME) arrived in separate cohorts on post-natal day (PND) 21 and were housed in same-sexed cages (4/cage) for 5 days in an AALAC-accredited facility under 12 h light/dark cycles. On PND 26, males and females were housed in same-sex groups in one of four conditions for the duration of the experiment: single housed (1/cage) with two bottles of water, single housed with one bottle ethanol and one bottle of water (i.e., two-bottle ethanol choice, 2-BC); neighbor housed (4/complex) with water or neighbor housed with 2-BC. Our custom-made neighbor housed cages were constructed from 4 standard polycarbonate ventilated cages (28.5 × 17.5 × 12.5 cm) with 4 circular cut-out ports (6 cm in diameter) along the sides conjoined by 4 polycarbonate portals [Figure 1, (Pais et al., 2019)]. Each portal was blocked with an 8 mm welded metal mesh with 1 cm^2^ wide openings that enabled limited physical interaction but full visual, olfactory and auditory stimuli from mice in the two conjoining cages. Thus, each neighbor-housed mouse had two same-sexed neighbors that remained constant. Each cage across housing condition was filled with woodchip bedding (Sani-Chips 7,090, Teklad/Envigo) and 1 square cotton nestlet (2.5 grams) was given at each cage change. Food (Teklad Diet 7,012) and water was present *ad libitum*. Neighbor housed and single housed mice resided in the same room and were exposed to similar olfactory, auditory, and visual stimuli. Experimenter interaction during this period of housing manipulation (from PND 26-106) was identical between groups and was restricted to body weight assessments every 2–3 days and weekly cage changes. One cohort of male (neighbor water *n* = 12; neighbor ethanol *n* = 12; single water, *n* = 8; single ethanol *n* = 16) and female (*n* = 12/group) animals were used for behavioral testing, which began on PND 54. A second cohort of male (*n* = 4–5/group) and female (*n* = 4–5/group) animals were housed and given access to ethanol or water as described above. On PND 57, PFC and NAc tissue was collected for microarray and qPCR analysis. All animal housing and care was conducted with the approval of the Virginia Commonwealth University IACUC Committee and in accordance with the NIH Guide for the Care and Use of Laboratory Animals (National Research Council (US) Committee for the Update of the Guide for the Care and Use of Laboratory Animals, 2011).

### 2.2 Two-bottle choice ethanol drinking

Home cage intermittent two-bottle choice drinking (2-BC) began on PND 29 for a total of 11 ethanol sessions in adolescence. Mice (*n* = 8–16/sex) were given intermittent access to two bottles, one containing ethanol (15% v/v) in tap water and the other containing tap water. A second group (*n* = 8–12/sex) were given two bottles of tap water in 10 mL conical tubes with rubber stoppers fitted with a ball-bearing sipper tube. Ethanol bottles were placed on cages on Mondays, Wednesdays, and Fridays on alternating sides at the beginning of the dark cycle for 24 h. Water and diet were present *ad libitum*. From PND 76-106, all adult groups were given 2-BC for ethanol to assess whether adolescent exposure increases adult intake and preference. Cages without animals but with water and ethanol bottles were used to correct for evaporation and bottle leakage. Ethanol intake and preference were calculated as gram ethanol intake per body weight and percent of ethanol volume divided by total liquid, respectively.

### 2.3 Social interaction test

On PND 54–55, 5 weeks after housing assignments, mice were habituated to the test room for 1 h and then tested for social interaction under low light conditions during the dark cycle. This task is a modified version of the social interaction task as previously described (Golden et al., 2011). Male and female mice (*n* = 8–16/group) were habituated to an open field locomotor activity box (41 × 41 × 31 cm, Omnitech Electronics, Inc.) containing an empty fine metal mesh cylinder for 3 min with no stimulus mouse present. The open field box was topographically divided into an interaction zone (25 × 7.5 cm) surrounding the stimulus mouse, and two corner zones (10 × 10 cm) far away from the interaction zone. Tracking software (Fusion v5.3; Omnitech Electronics Inc.) was used to record movement and animal position via infrared photobeam breaks. Between the habituation and test, mice were returned to their home cage for 30 s, while an unfamiliar adult female C57BL/6J mouse was placed under the inverted fine metal mesh cylinder (8.5 cm diameter, 11 cm height). During the test phase, the amount of time and distance the test mouse traveled in the interaction zone or in the opposing corner zones was recorded for 3 min. Three mice were excluded because the stimulus mouse escaped the cylinder during the test.

### 2.4 Novel object recognition

We used the novel object recognition task to measure PFC-mediated recognition memory, as previously described (Wolstenholme et al., 2017). Novel object recognition involved a training and a test phase, separated by a 5 min inter-trial interval. On PND 56–58, mice (*n* = 8–16/group) were habituated to the test cage for 30 min one day prior to the task. On test day, mice were habituated to the testing room for one hour, then to the test cage for 30 min. During the training phase, mice were allowed to interact with two identical objects, placed in opposite corners of an empty clean mouse cage, for 5 min. Mice were then returned to their home cage for a 5 min inter-trial delay to measure PFC-dependent short term memory (Warburton and Brown, 2015). During the inter-trial delay, one familiar object was replaced by a novel object of similar size. Mice were returned to the test arena and allowed to explore both objects for 5 min. Time in close contact (<2 cm) with nose oriented towards the object was recorded. Scorers were blinded to the sex and treatment of the mice. A discrimination index was calculated by subtracting the time interacting with the familiar object from the time interacting with the novel object divided by the total interaction time. Failure to spend more time with the novel object was interpreted as impaired recognition memory and PFC dysfunction (Weitzel et al., 2015). Any mouse that did not investigate the objects for more than 10 s during training was not used in the analysis. No mice were excluded for this reason.

### 2.5 Anxiety-like behavior in the light–dark box

At PND 64, mice (*n* = 8–16/group) were tested for differences in basal anxiety-like behavior during the dark phase of the light–dark cycle. The light–dark box conflict model for anxiety-like behavior was conducted using the Fusion tracking software (Fusion v5.3; Omnitech Electronics Inc.) to record movement in an open field activity box divided into two equally sized light and dark zones (25.4 × 12.7 × 20.3 cm), as previously described (Wolstenholme et al., 2017; Pais et al., 2019). Animal position and locomotor activity was monitored by infrared photobeam breaks. Following a 1 h acclimation period to the behavioral room, mice were placed in the center of the light chamber facing the entrance to the dark chamber. Studies consisted of a 5 min test session, initiated once the animal entered the dark compartment. Measures recorded were percent time spent in the light and percent distance traveled in the light. An increase in either measure was interpreted as decreased anxiety-like behavior. The total distance travelled in the arena was also recorded as a measure of locomotor activity. One female mouse was excluded from this analysis due to a lack of any locomotor activity.

### 2.6 RNA isolation and microarray analysis

Adolescent mice for these studies were run concurrently with the male and female behavioral cohorts above. Mice (*n* = 4–5/group) were single or neighbor housed on PND 26. Half of the mice were given ethanol 2-BC drinking and half the mice were given two bottles of water from PND 29–52, as above. PFC and NAc were collected on PND 57 (*n* = 4–5/group) and flash frozen. RNA was isolated using STAT 60 Reagent (Tel-Test, Friendswood, TX, United States) and RNeasy mini kit (Qiagen, Valencia, CA, United States) according to the manufacturer’s protocol. RNA concentration was determined by absorbance at 260 nm and RNA quality was assessed by Experion automated electrophoresis (Bio-Rad, Hercules, CA, United States) and 28S:18S ratios. All RNA RQI values were >9.0, and 260/280 ratios were between 1.9 and 2.1. PFC and NAc RNA was reverse transcribed and labeled for microarray hybridization using standard kits and protocols from Affymetrix as described (Wolstenholme et al., 2011, 2017). Labeled cDNA was hybridized to GeneChip Mouse Transcriptome Arrays (ClariomS; *n* = 78). Each array was processed through quality control, normalization using Expression Console and the Transcriptome Analysis Center (TAC, Affymetrix), and bioinformatics pipelines previously established (Wolstenholme et al., 2011, 2017). One microarray (one single housed ethanol female PFC) failed quality control checks, displaying low signal intensity suggesting poor hybridization and was not used in this analysis. Differential gene expression was determined using signal space transformation RMA (sstRMA) generated by the Expression Console (Affymetrix, Santa Clara, CA, United States). All arrays were run simultaneously but differential expression analysis was performed separately for each sex and brain region. Significant differentially expressed gene (DEG) lists were generated using a two-way ANOVA (for the factors of housing and drinking) in the Transcriptome Analysis Center (TAC) software (Affymetrix). Significant differentially expressed transcript IDs were called at uncorrected *p* < 0.05 and fold change +/− 1.2. Full gene sets can be found in Supplementary Tables S1–S2.

Bioinformatics analysis was performed using previously established pipelines (Wolstenholme et al., 2011, 2013, 2017) and included functional over-representation analysis with Gene Ontology (GO) using the ToppFun suite of tools (Chen et al., 2009) in the ToppGene Suite for gene list enrichment analysis. Differentially expressed gene lists were subjected to gene ontology analysis if there were >50 genes in the list. Gene sets were filtered based on the number of genes within each category (min = 4, max = 500) and *p* < 0.05. Lists were further filtered by gene list hits, where categories containing <3 hits were excluded. Categories that had identical query gene lists and similar category names were removed to reduce repetitiveness. The top 15 molecular function and biological process categories are represented in our figures. For analyses with ≤100 differentially expressed genes, the top 5 molecular function and biological process categories are represented. Full gene ontology results can be found in Supplementary Tables S1–S2.

### 2.7 Quantitative real-time PCR

To confirm the microarray findings on candidate genes, PFC and NAc total RNA from a was reverse transcribed to cDNA using the iScript cDNA kit (Bio-Rad, Hercules, CA, United States). Real-time PCR was performed using the CFX System (Bio-Rad) for SYBR Green-based detection using standard protocols (Wolstenholme et al., 2011, 2012, 2013, 2017). Biological replicate samples (*n* = 4/group) were run in triplicate. Quantification of candidate gene expression levels was calculated based on the threshold cycle (Ct) for each well using the provided software and normalized to *PPP2r2p* and *Ublcp1* as endogenous controls. Relative changes in gene expression were normalized to the control male group.

### 2.8 Statistics

Behavioral and qPCR data was analyzed using two-way ANOVAs with housing condition and ethanol drinking as factors. Ethanol 2-BC intake and preference were analyzed using repeated measures two-way ANOVAs with housing and day as factors. We analyzed male and female data separately as we were interested in behavioral and gene expression changes within each sex. These cohorts were also run separately for this reason and due to space constraints. Tukey’s *post hoc* tests were used to calculate significance. *p*-values less than 0.05 were considered significant.

## 3 Results

### 3.1 Adolescent social isolation decreases social interaction in adulthood

**Figure 2:**
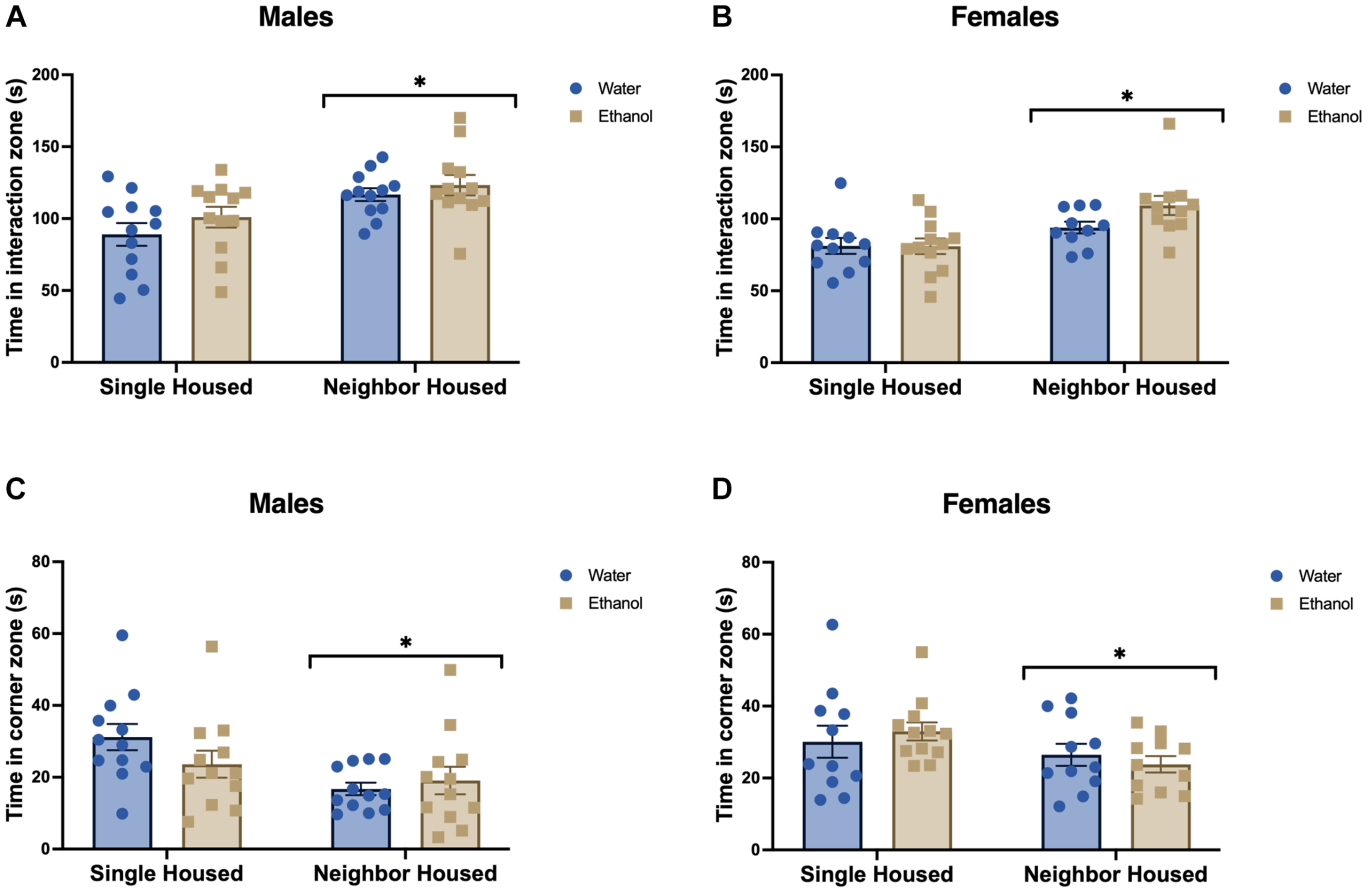
Adolescent social isolation decreases social interaction in males and females. Time spent in the interaction zone with a stimulus mouse was significantly increased in neighbor housed mice as compared with group housed mice in males **(A)** and females **(B)**. Time spent in the corner zones far away from the stimulus mouse was significantly increased due to single housing in males **(C)** and females **(D)**. Ethanol drinking did not alter the behavior of males or females in either the time in the interaction or corner zones. *p < 0.05 by two-way ANOVA.

After 5 weeks of single or neighbor housing +/− ethanol 2-BC, mice were tested in the social interaction task towards a novel female stimulus mouse to assess social approach and social anxiety-like behavior (Golden et al., 2011). Single housed males, regardless of ethanol drinking history, spent less time in the interaction zone (*F*_housing_ = 13.56, *p* = 0.0006, Figure 2A) and more time in the corner zones, far away from the stimulus mouse (*F*_housing_ = 7.97, *p* = 0.007, Figure 2C). Ethanol drinking did not alter the behavior of single or neighbor housed males in either the time in the interaction zone (*F*_drinking_ = 1.88, *p* = 0.177, Figure 2A) or the time in the corner zones (*F*_drinking_ = 0.60, *p* = 0.441, Figure 2C). No significant interactions were found (interaction zone: *F*_interaction_ = 0.158, *p* = 0.693; corner zones: *F*_interaction_ = 2.16, *p* = 0.149).

Similarly, in females, single housed mice spent less time interacting with a novel female stimulus as compared to neighbor housed females (*F*_housing_ = 13.50, *p* = 0.0007, Figure 2B). Time in the corner zones, far away from the stimulus mouse, was greater in single housed mice than neighbor housed (*F*_housing_ = 4.08, *p* = 0.049, Figure 2D). As seen with the males, ethanol drinking in adolescence did not further impact the time engaging with a novel stimulus mouse (*F*_drinking_ = 1.87, *p* = 0.180, Figure 2B) or time in the corner zones (*F*_drinking_ = 0.0014, *p* = 0.9702, Figure 2D). No significant interactions were found in the interaction zone (*F*_interaction_ = 1.96, *p* = 0.169) or in the corner zones (*F*_interaction_ = 0.758, *p* = 0.389).

### 3.2 Social isolation in adolescence leads to cognitive deficits in adulthood

**Figure 3:**
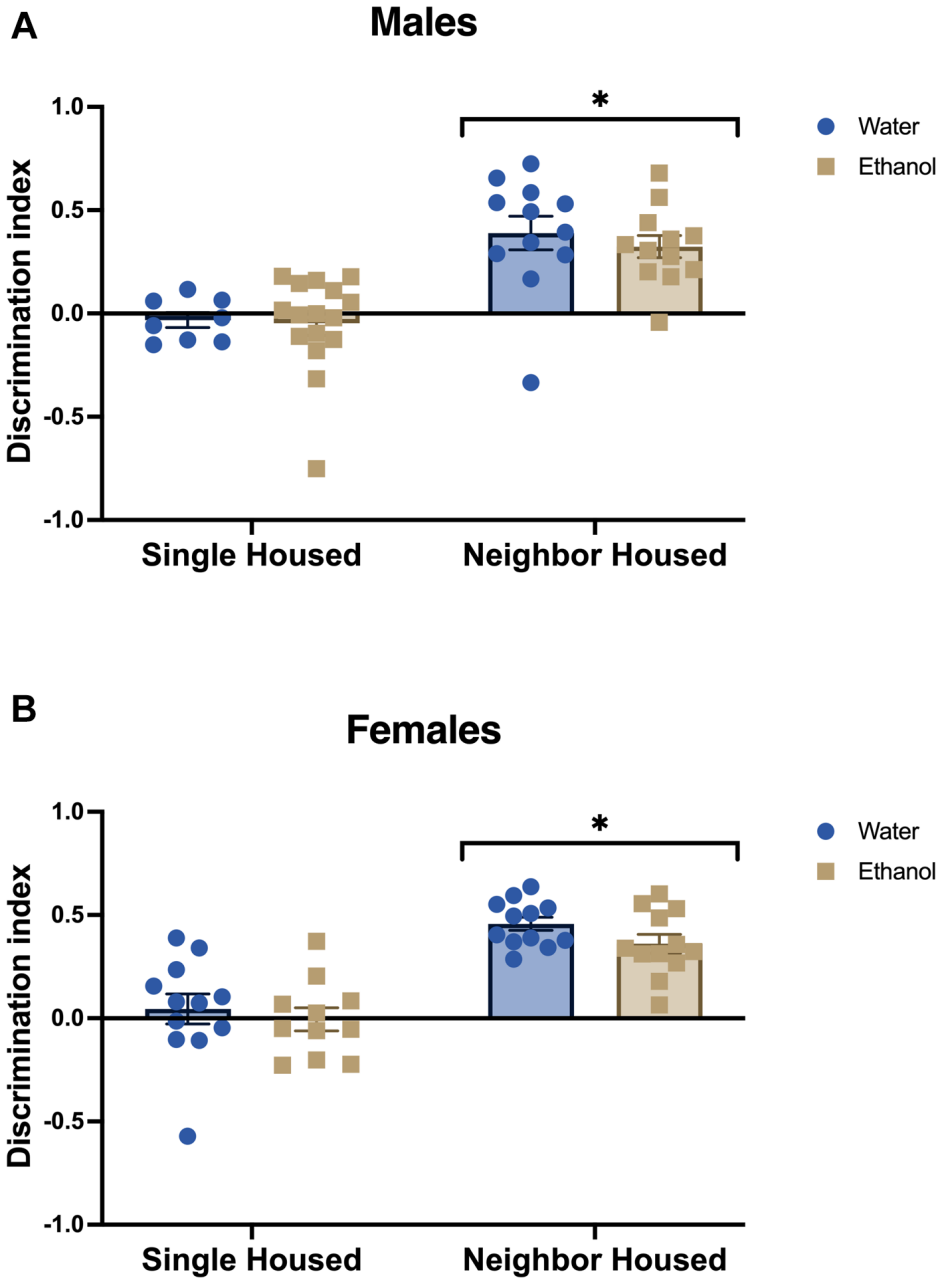
Adolescent social isolation induces memory deficits in males and females. In the novel object recognition task, single housed mice had a lower discrimination index as compared to male **(A)** and female **(B)** neighbor housed mice. Ethanol drinking did not alter the behavior of males or females. *p < 0.05 by two-way ANOVA.

Similar to our previous findings (Pais et al., 2019), single housing in adolescent male and female mice led to deficits in novel object recognition in adulthood. The discrimination index for the novel object was significantly lower in single housed males (*F*_housing_ = 36.17, *p* < 0.0001, Figure 3A) and females (*F*_housing_ = 52.76, *p* < 0.0001, Figure 3B). A history of drinking in adolescence did not impact performance in the novel object recognition task in either males (*F*_drinking_ = 0.38, *p* = 0.541, Figure 3A) or females (*F*_drinking_ = 1.87, *p* = 0.179, Figure 3B). No significant interactions between housing and ethanol drinking were found in males (*F*_interaction_ = 0.142, *p* = 0.708) or females (*F*_interaction_ = 0.186, *p* = 0.669).

### 3.3 Neighbor housing reduces anxiety-like behavior in the light–dark box

**Figure 4:**
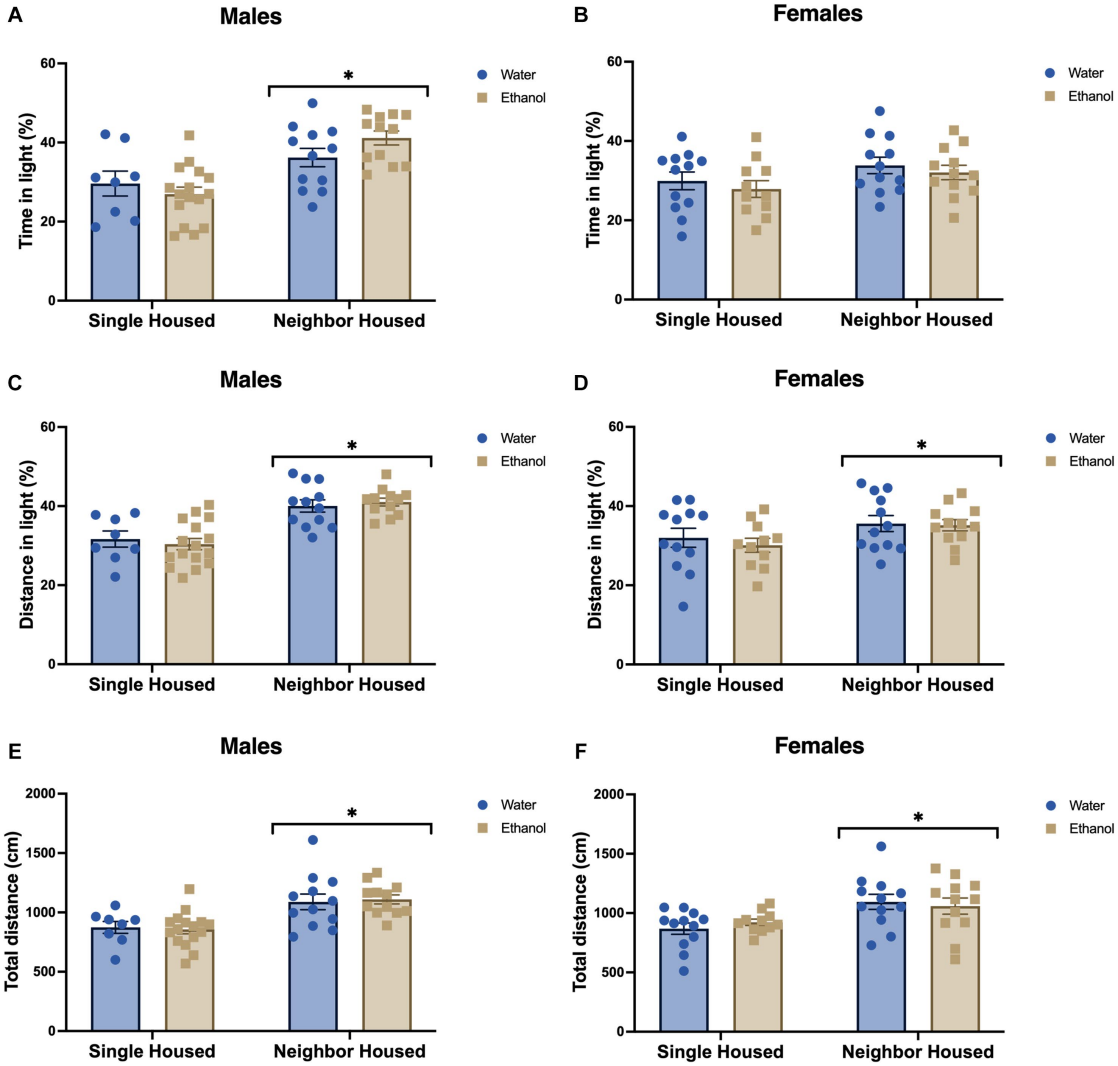
Neighbor housing reduces anxiety-like behavior in the light–dark box. In male animals, percent time in the light was significantly increased in neighbor housed males **(A)**, but only showed a trend for an increase in females **(B)** as compared to single housed animals. Distance in the light **(C,D)**, and total distance travelled **(E,F)** were significantly increased in neighbor housed mice as compared to single housed mice in both males and females. An adolescent history of ethanol drinking did not influence these behaviors in males or females. *p < 0.05 by two-way ANOVA.

Since social anxiety-phenotypes were seen in single housed mice in the social interaction task, we also assessed basal anxiety-like behavior in the light–dark conflict model. Single housed mice displayed higher anxiety-like phenotypes in the light–dark box. Single housed males spent a lower percent of the total time (*F*_housing_ = 21.92, *p* < 0.0001, Figure 4A) and traveled a lower percent of the total distance (*F*_housing_ = 38.56, *p* < 0.0001, Figure 4C) in the light section of the arena as compared to neighbor housed mice. Again, an adolescent history of ethanol drinking did not influence this behavior in males. Percent time in the light (*F*_drinking_ = 0.26, *p* = 0.613, Figure 4A) and percent distance in the light (*F*_drinking_ = 0.008, *p* = 0.928, Figure 4C) did not differ due to a history of drinking. No significant interactions between housing and drinking were found (percent time in light: *F*_interaction_ = 2.95, *p* = 0.093; percent distance in light: *F*_interaction_ = 0.542, *p* = 0.466).

In contrast to male single housed findings, single housed females had a trend for higher percent time in the light (*F*_housing_ = 3.81, *p* = 0.057, Figure 4B), and a significantly greater percent distance traveled in the light (*F*_housing_ = 4.95, *p* = 0.031, Figure 4D). In congruence with our ethanol-treated males, a history of ethanol drinking in adolescence did not affect the percent time (*F*_drinking_ = 0.861, *p* = 0.359, Figure 4B) or the percent distance (*F*_drinking_ = 0.36, *p* = 0.555, Figure 4D) in the light in female animals. No significant interactions between housing and drinking were found (percent time in light: *F*_interaction_ = 0.005, *p* = 0.945; percent distance in light: *F*_interaction_ = 0.136, *p* = 0.714).

Notably, both neighbor housed males and females displayed increased total locomotor activity in the light–dark arena. A main effect of housing was found in the males (*F*_housing_ = 22.07, *p* < 0.0001, Figure 4E) and in the females (*F*_housing_ = 10.90, *p* = 0.002, Figure 4F), where neighbor mice traveled farther in the arena than single housed mice. A history of ethanol drinking did not impact this metric (males: *F*_drinking_ = 0.002, *p* = 0.965, Figure 4E; females: *F*_drinking_ = 0.029, *p* = 0.886, Figure 4F). No significant interactions were found for the total distance traveled in the light–dark box in males (*F*_interaction_ = 0.141, *p* = 0.709) or females (*F*_interaction_ = 0.688, *p* = 0.412).

### 3.4 Social isolation does not alter ethanol drinking as compared to neighbor housed mice

**Figure 5:**
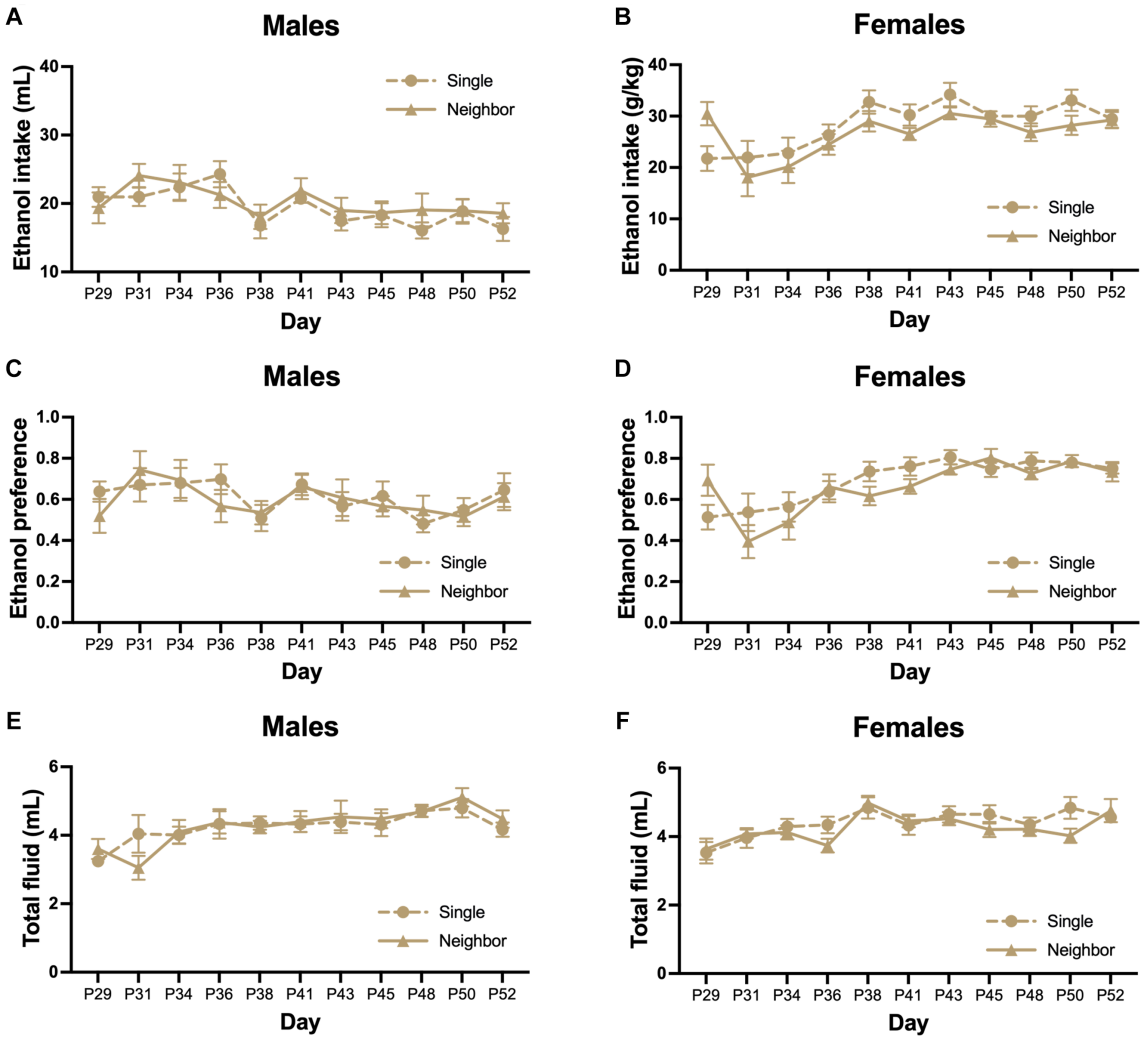
Adolescent social isolation does not alter ethanol drinking as compared to neighbor housed mice. In adolescence, ethanol consumption was measured using an intermittent access 2-BC paradigm where animals were given the choice between one bottle of ethanol (15% v/v) in tap water or tap water alone. Control groups were given two bottles of tap water (not shown). Mice were housed in single cages or in neighbor cages from PND 29–52 and we compared ethanol intake, preference, and total fluid between groups. There was no significant effect of housing on adolescent ethanol intake **(A,B)**, preference **(C,D)**, or total fluid consumption **(E,F)** in males or females.

In adolescence from PND 29–52, we measured ethanol consumption using an intermittent access 2-BC paradigm or access to two bottles of water. In males, there was no significant effect of housing on adolescent ethanol intake (*F*_housing_ = 0.218, *p* = 0.646, Figure 5A), preference (*F*_housing_ = 0.172, *p* = 0.683, Figure 5C), or total fluid consumption (*F*_housing_ = 0.056, *p* = 0.814, Figure 5E). We did find that there was an effect of postnatal day on ethanol intake (*F*_day_ = 4.68, *p* < 0.0001, Figure 5A), preference (*F*_day_ = 1.91, *p* = 0.047, Figure 5C) and total fluid (*F*_day_ = 4.08, *p* < 0.0001, Figure 5E). No significant interactions between day and housing condition were found for ethanol intake (*F*_interaction_ = 0.859, *p* = 0.573), preference (*F*_interaction_ = 0.508, *p* = 0.883), or total fluid (*F*_interaction_ = 0.440, *p* = 0.925). Body weight increased over the course of the study (*F*_day_ = 1,060, *p* < 0.0001, Supplementary Figure S1A), but did not differ between housing conditions (*F*_housing_ = 0.99, *p* = 0.400) or the interaction between housing and day (*F*_interaction_ = 1.48, *p* = 0.092).

In females, housing condition did not alter adolescent ethanol intake (*F*_housing_ = 1.45, *p* = 0.243, Figure 5B), ethanol preference (*F*_housing_ = 0.672, *p* = 0.422, Figure 5D) or total fluid consumption (*F*_housing_ = 0.813, *p* = 0.378, Figure 5F). Overall, there was a significant increase over postnatal day for ethanol intake (*F*_day_ = 7.178, *p* < 0.0001, Figure 5B), ethanol preference (*F*_day_ = 8.69, *p* < 0.0001, Figure 5D), and total fluid (*F*_day_ = 5.433, *p* < 0.0001, Figure 5F). No significant interactions between day and housing condition were found for ethanol intake (*F*_interaction_ = 1.52, *p* = 0.135), preference (*F*_interaction_ = 1.48, *p* = 0.149), or total fluid (*F*_interaction_ = 1.19, *p* = 0.303). Body weight increased over the course of the study (*F*_day_ = 329, *p* < 0.0001, Supplementary Figure S1B), but did not differ between housing conditions (*F*_housing_ = 0.090, *p* = 0.965) or the interaction between housing and day (*F*_interaction_ = 1.03, *p* = 0.421).

### 3.5 A history of adolescent ethanol drinking does not alter adult ethanol intake

**Figure 6:**
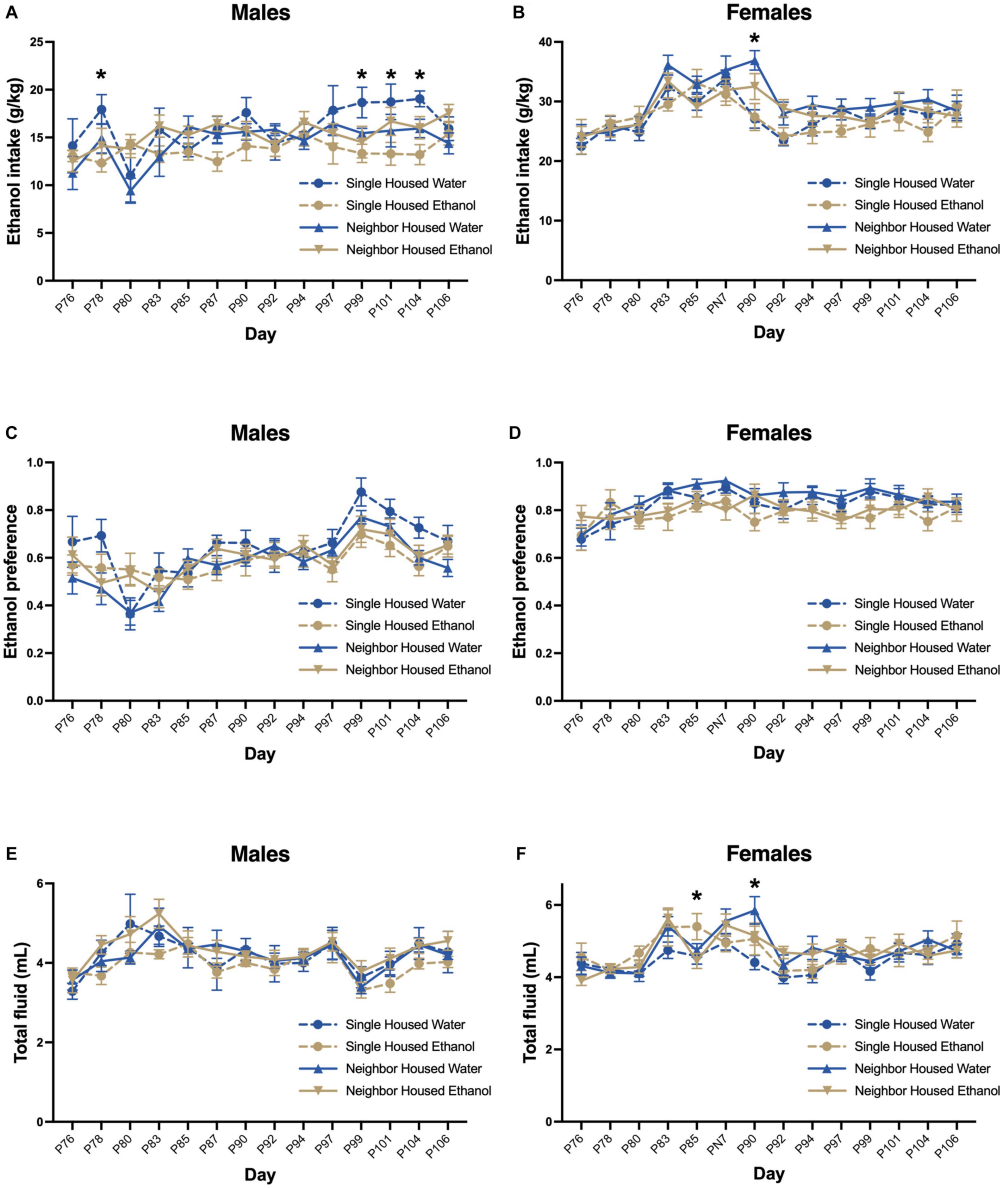
A history of ethanol drinking does not alter adult intake. In adulthood, all groups were given 2-BC for ethanol to assess whether adolescent exposure increases adult intake and preference from PND 76–106. Two-way RMANOVA revealed no significant effect of housing on ethanol intake **(A,B)**, preference **(C,D)**, or total fluid consumption **(E,F)** in males or females. Significant interactions were found for ethanol intake in males **(A)** on PND 78, 99, 101, and 104 (single water > neighbor water) and in females **(B)** on PND 90 (single water < neighbor water). Total fluid consumed also differed in females **(F)** by housing and day on PND 85 (single ethanol > neighbor ethanol) and PND 90 (single water < neighbor water). *p < 0.05 by two-way RMANOVA.

In adulthood, all groups were given 2-BC for ethanol to assess whether adolescent exposure increases adult intake and preference. There was no effect of housing on ethanol drinking behavior in adult mice. In males, two-way RMANOVA revealed no significant effect of housing or history of ethanol on ethanol intake (*F*_group_ = 2.74, *p* = 0.056, Figure 6A), preference (*F*_group_ = 1.48, *p* = 0.236, Figure 6C), or total fluid (*F*_group_ = 1.02, *p* = 0.392, Figure 6E). There was a significant effect of postnatal day on ethanol intake (*F*_day_ = 3.82, *p* < 0.0001, Figure 6A), preference (*F*_day_ = 12.47, *p* < 0.0001, Figure 6C) and total fluid (*F*_day_ = 9.13, *p* < 0.0001, Figure 6E). Significant interactions in ethanol intake were found between housing, prior ethanol intake and day (*F*_interaction_ = 1.45, *p* = 0.043), where adolescent single housed water drinkers consumed more ethanol as adults than single housed ethanol drinkers on days 78, 99, 101, and 104 (by Tukey post-hoc, *p* < 0.05). Neighbor housed males with a history of ethanol drinking did not appear to escalate their ethanol intake in the last few days of access (in weeks 4–5). Conversely, neighbor housed males with no ethanol drinking history escalated their intake on these days. Ethanol preference (*F*_interaction_ = 1.40, *p* = 0.060) and total fluid (*F*_interaction_ = 0.807, *p* = 0.793) did not show significant interactions between housing or day. Body weight increased over the course of the study (*F*_day_ = 93.9, *p* < 0.0001, Supplementary Figure S1C), but did not differ between group (*F*_group_ = 0.584, *p* = 0.629). A significant interaction between housing and day was found (*F*_interaction_ = 1.94, *p* = 0.034), but post-hoc tests were not significant.

There was no significant effect of housing or history of drinking on ethanol intake (*F*_group_ = 0.127, *p* = 0.296, Figure 6B), preference (*F*_group_ = 0.166, *p* = 0.190, Figure 6D), or total fluid consumption (*F*_group_ = 0.811, *p* = 0.495, Figure 6F) in females. There was an effect of postnatal day on ethanol intake (*F*_day_ = 17.36, *p* < 0.0001, Figure 6B), preference (*F*_day_ = 5.02, *p* < 0.001, Figure 6D) and total fluid (*F*_day_ = 13.17, *p* < 0.0001, Figure 6F) in adulthood. There was also a significant interaction between postnatal day, housing and prior ethanol drinking for adult intake (*F*_interaction_ = 1.81, *p* = 0.0024, Figure 6B) and total fluid (*F*_interaction_ = 2.12, *p* = 0.001, Figure 6D), but not preference (*F*_interaction_ = 1.09, *p* = 0.325, Figure 6F). On PND 90, single housed females that consumed water only in adolescence had lower ethanol intake and lower total fluid intake as compared to neighbor housed females that consumed water in adolescence. Total fluid intake was also slightly increased on PND 85 in single housed ethanol drinking females as compared to neighbor housed ethanol drinking females. Body weight increased over the course of the study (*F*_day_ = 147.4, *p* < 0.0001, Supplementary Figure S1D), but did not differ between group (*F*_group_ = 1.24, *p* = 0.307) or the interaction between group and day (*F*_interaction_ = 0.729, *p* = 0.722).

### 3.6 Differentially expressed genes in the PFC

**Figure 7:**
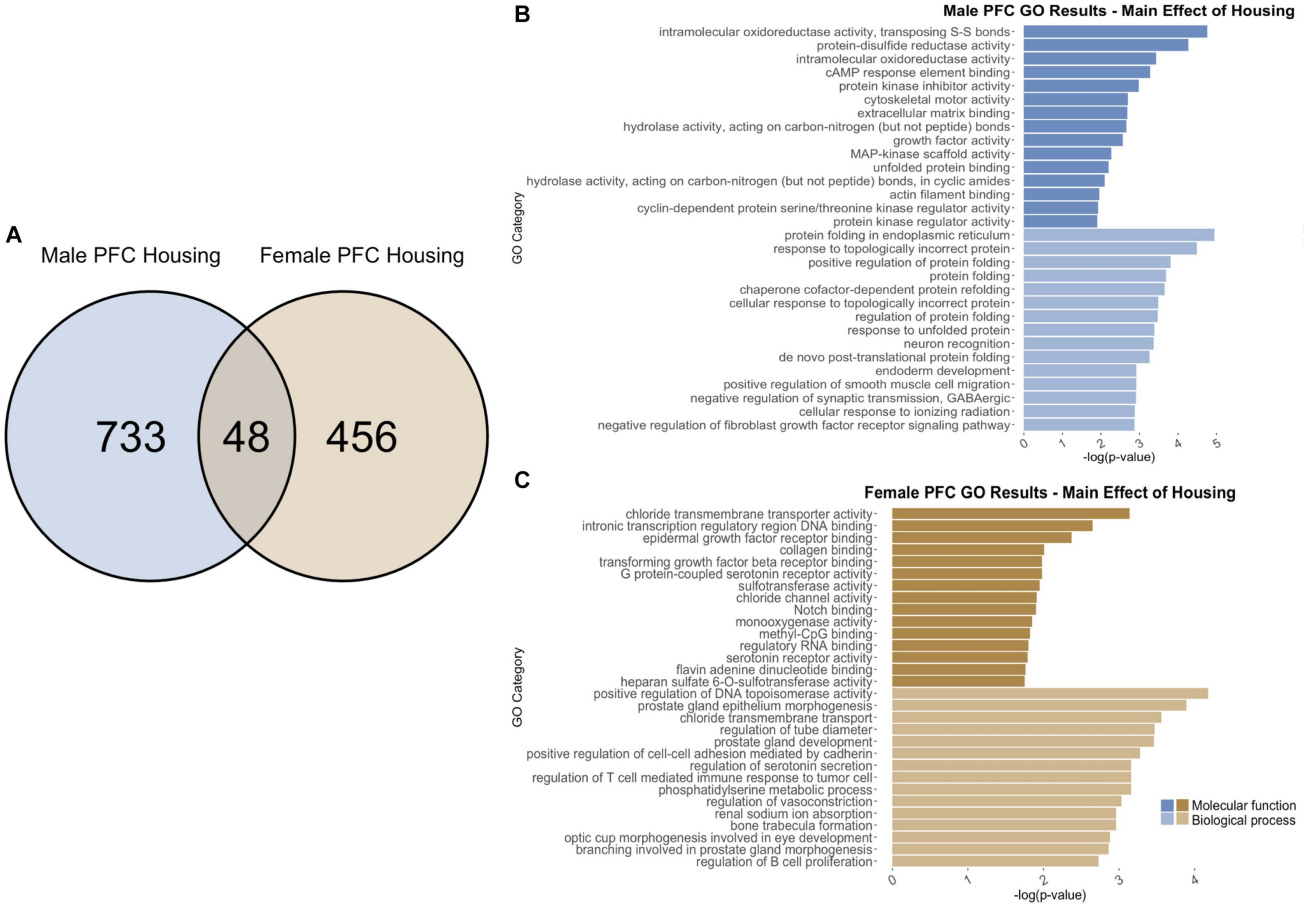
GO analysis showing genes categories differentially impacted by housing condition in the PFC. **(A)** Number of DEGs due to housing condition in male and female PFC at *p* < 0.05. **(B)** GO analysis of differentially expressed genes unique to males. **(C)** GO analysis of differentially expressed genes unique to females.

**Figure 8:**
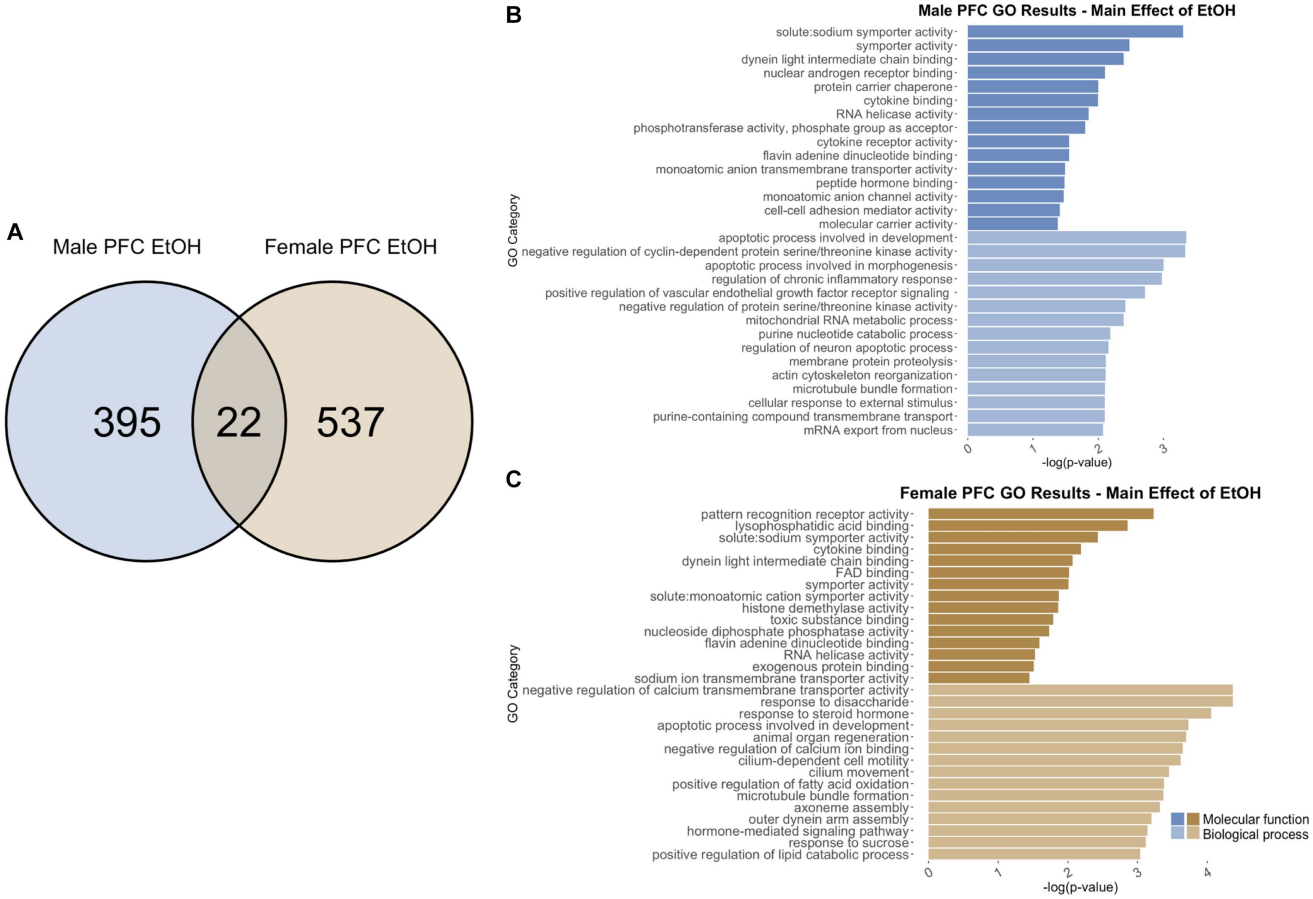
GO analysis showing genes categories differentially impacted by ethanol treatment in the PFC. **(A)** Number of DEGs due to ethanol treatment in male and female PFC at *p* < 0.05. **(B)** GO analysis of differentially expressed genes unique to males. **(C)** GO analysis of differentially expressed genes unique to females.

**Figure 9:**
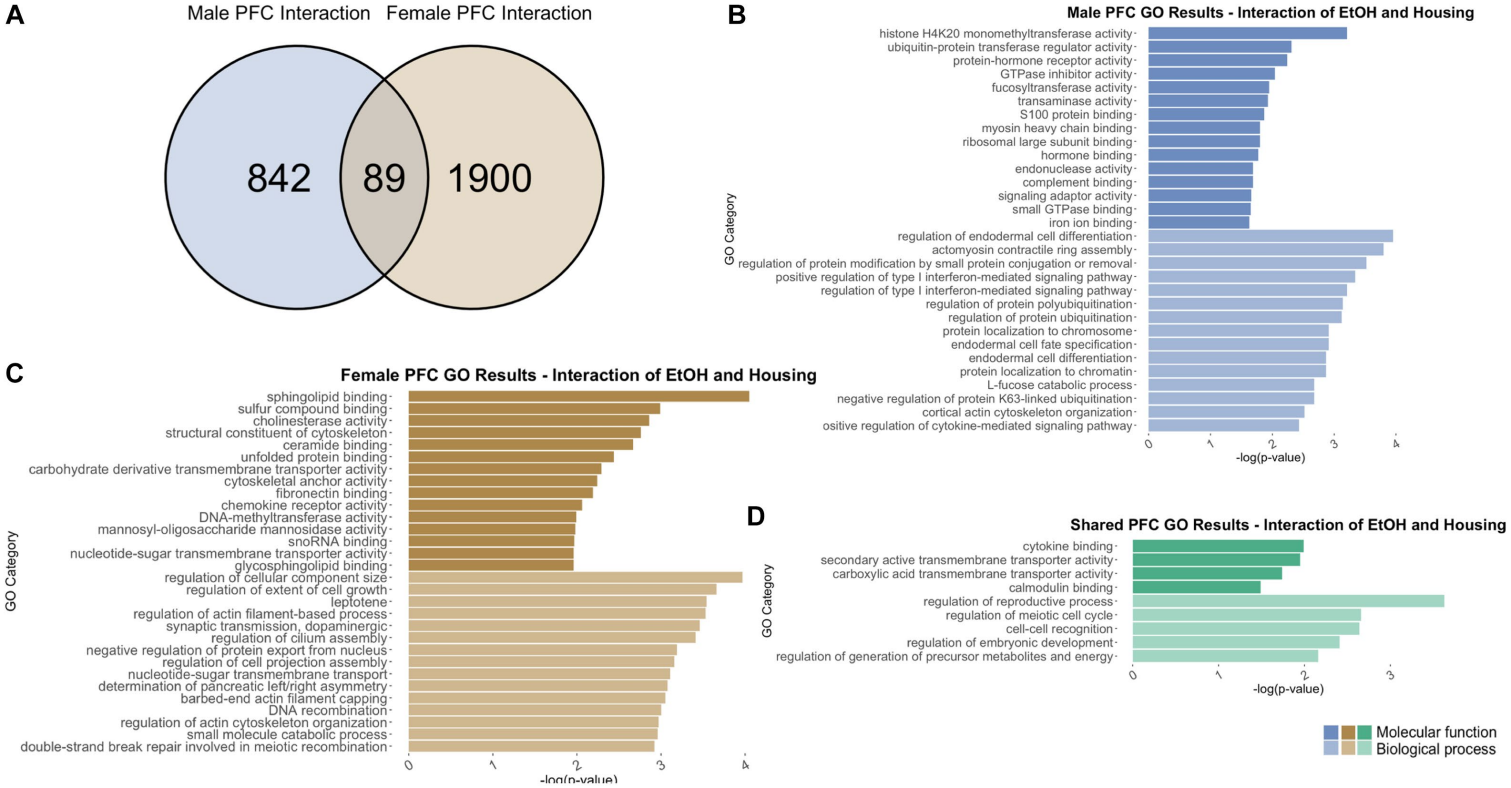
GO analysis showing genes categories differentially impacted by the interaction of housing condition and ethanol treatment in the PFC. **(A)** Number of DEGs due to the interaction of housing condition and ethanol treatment in male and female PFC at *p* < 0.05. **(B)** GO analysis of differentially expressed genes unique to males. **(C)** GO analysis of differentially expressed genes unique to females. **(D)** GO analysis of differentially expressed genes in both males and females due to the interaction of housing condition and ethanol treatment.

**Figure 10:**
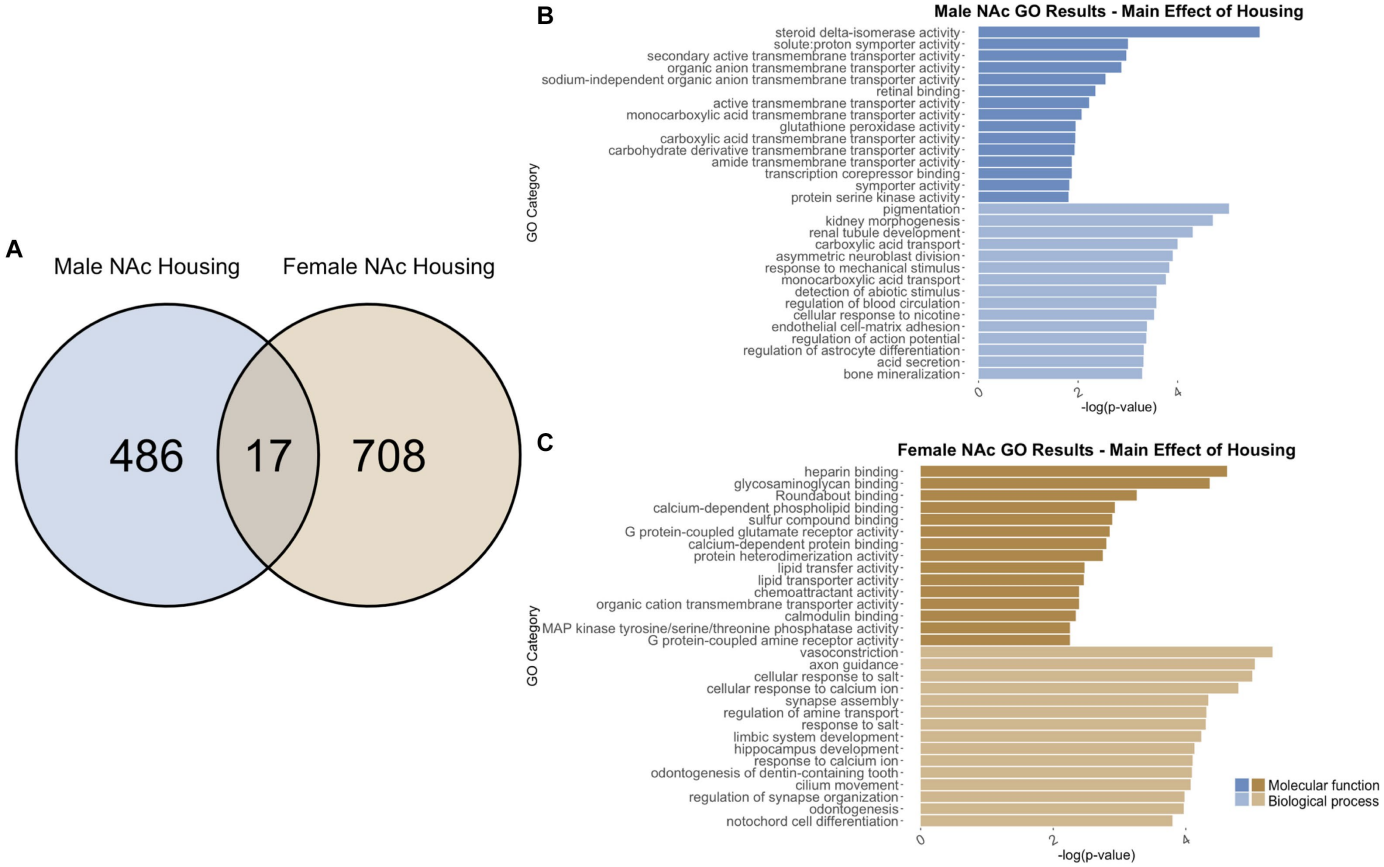
GO analysis showing genes categories differentially impacted by housing condition in the NAc. **(A)** Number of DEGs due to housing condition in male and female NAc at *p* < 0.05. **(B)** GO analysis of differentially expressed genes unique to males. **(C)** GO analysis of differentially expressed genes unique to females.

**Figure 11:**
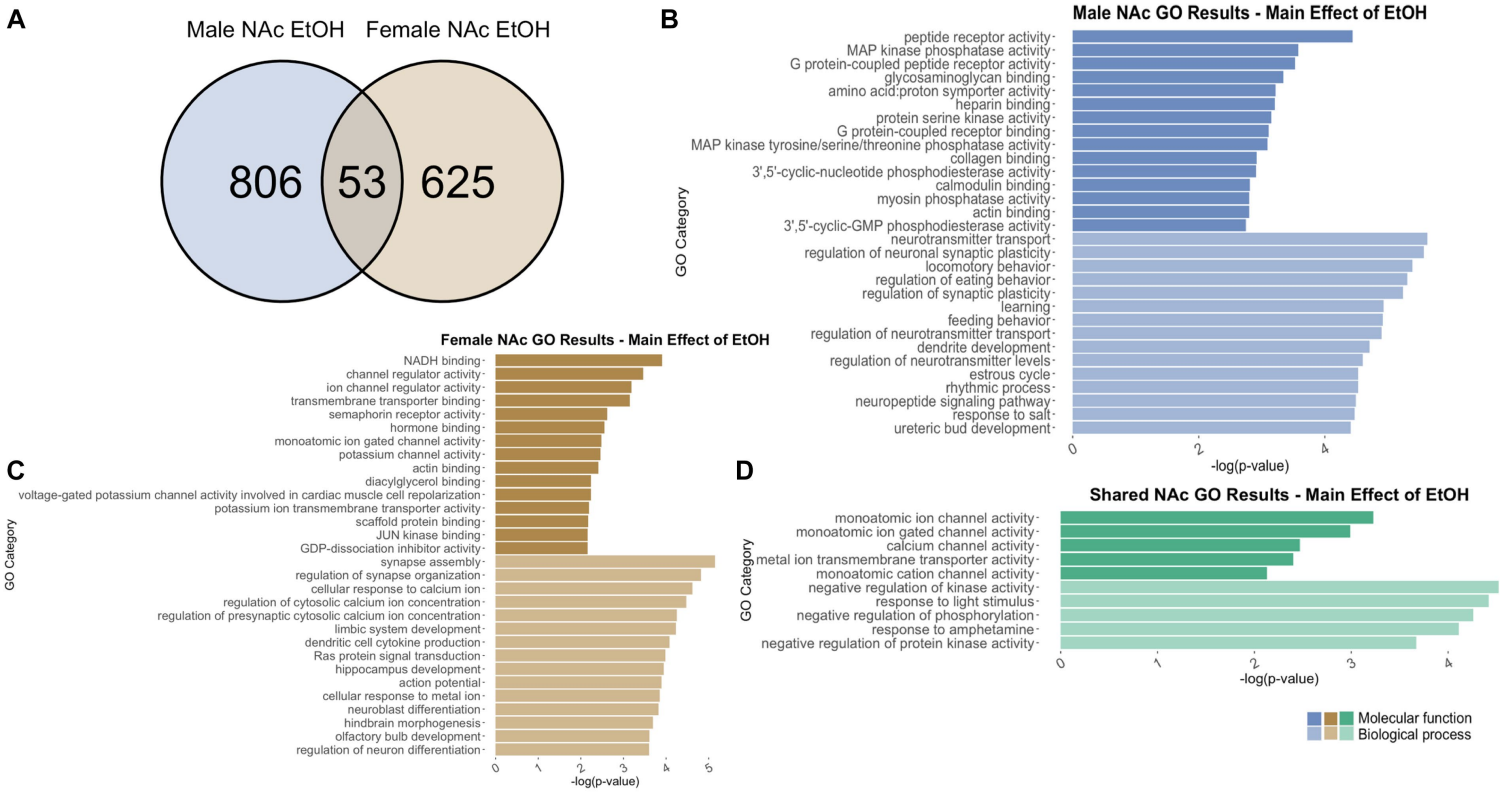
GO analysis showing genes categories differentially impacted by ethanol treatment in the NAc. **(A)** Number of DEGs due to ethanol treatment in male and female NAc at *p* < 0.05. **(B)** GO analysis of differentially expressed genes unique to males. **(C)** GO analysis of differentially expressed genes unique to females.

**Figure 12:**
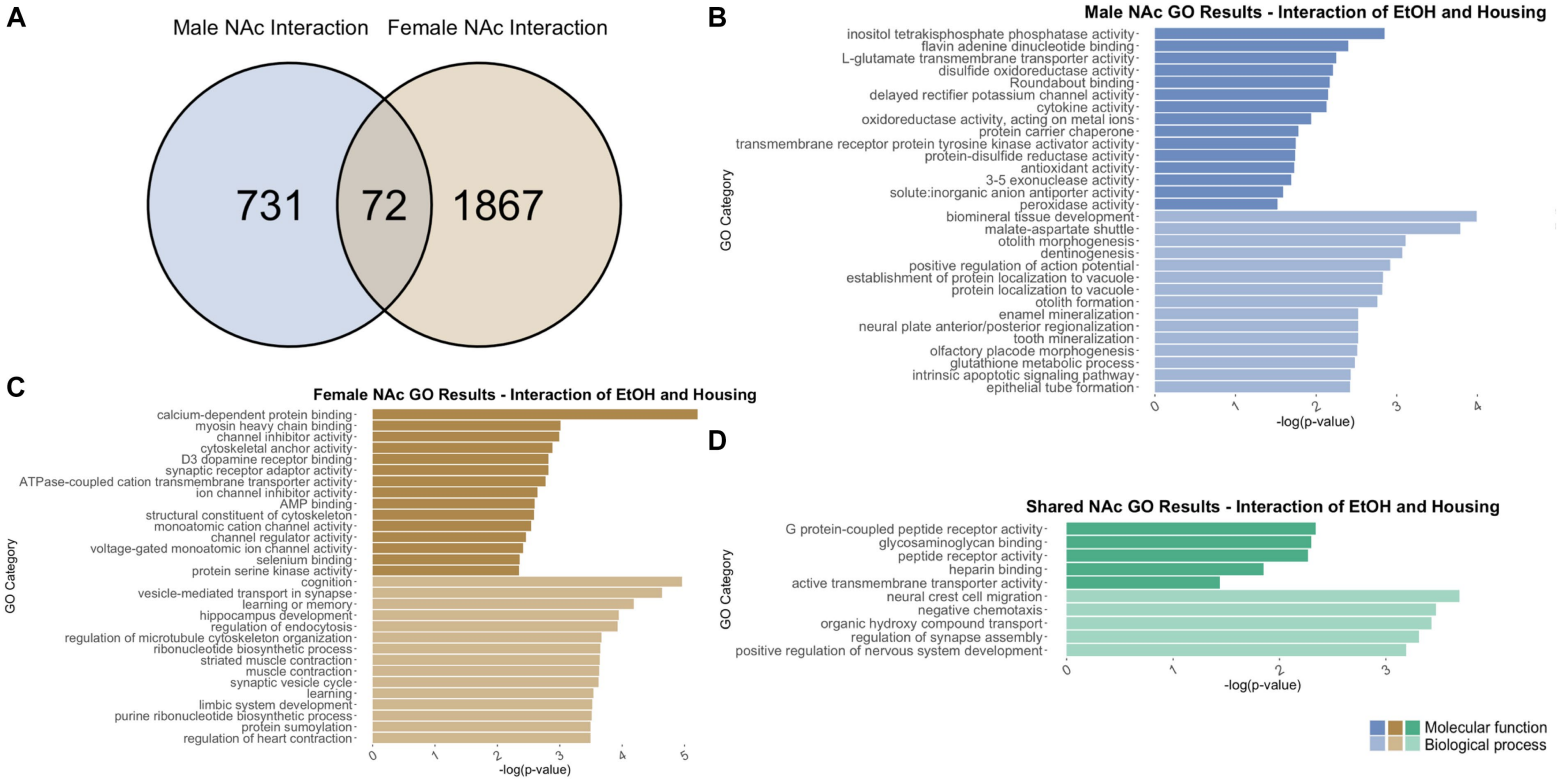
GO analysis showing genes categories differentially impacted by the interaction of housing condition and ethanol treatment in the NAc. **(A)** Number of DEGs due to the interaction of housing condition and ethanol treatment in male and female NAc at *p* < 0.05. **(B)** GO analysis of differentially expressed genes unique to males. **(C)** GO analysis of differentially expressed genes unique to females. **(D)** GO analysis of differentially expressed genes in both males and females due to the interaction of housing condition and ethanol treatment.

To identify genes that were differentially regulated by adolescent ethanol exposure, single housing conditions, or showed an interaction between ethanol exposure and housing conditions, we defined differential expression in the microarray data to include transcripts with a value of *p* <0.05 and a|fold change| > 1.2 to generate a gene list of sufficient length for gene discovery using downstream gene ontology analyses. Full gene ontology results can be found in Supplementary Tables S1–S2. The first 15 molecular function and biological process categories were selected to represent our results in Figures 7–12 although if gene lists had <50 genes, only the top 5 molecular function and biological process categories were selected to represent our results.

#### 3.6.1 PFC differentially expressed genes due to housing

Comparing single housing versus neighbor housing conditions throughout adolescence identified a total of 781 differentially regulated genes in the PFC of males (Supplementary Table S1). Of those, 733 genes were unique to males (Figure 7A) and served as the input for male-specific gene ontology over-representation analysis using ToppFun. Results of the gene ontology analysis are shown in Figure 7B. The top significant categories for molecular function included protein kinase inhibitor activity and extracellular matrix binding. The top significant categories for biological process included protein folding and negative regulation of synaptic transmission, GABAergic.

In females, 504 were differentially expressed in single versus neighbor housing (Supplementary Table S1). Of those, 456 genes were unique to females and served as the input for female-specific gene ontology over-representation analysis using ToppFun (Figure 7A). The top significant categories for molecular function included transforming growth factor beta receptor binding, methyl CpG binding, and serotonin receptor activity. The top significant categories for biological process included positive regulation of cell–cell adhesion by cadherin and regulation of serotonin secretion (Figure 7C).

781 genes were differentially expressed in males, and 504 genes were differentially expressed in females due to single versus neighbor housing throughout adolescence. Of those, only 48 genes were differentially expressed in both males and females (Figure 7A), and gene ontology analysis was not run due to the low number of genes. However, several immediate-early genes were found to be differentially expressed due to single housing in both males and females including *Arc*, *Egr1*, and *Egr3*.

#### 3.6.2 PFC differentially expressed genes due to adolescent ethanol

Adolescent ethanol exposure differentially regulated 422 total genes in the PFC of males (Supplementary Table S1) as compared to water drinking males. Of those, 417 genes were unique to males (Figure 8A) and served as the input for male-specific gene ontology over-representation analysis using ToppFun. Results of the gene ontology analysis are shown in Figure 8B. The first 15 molecular function and biological process categories were selected to represent our results. The top significant categories for molecular function included cytokine binding, RNA helicase activity, and cell–cell adhesion mediator activity. The top significant categories for biological process included regulation of chronic inflammatory response, regulation of neuron apoptotic process, and actin cytoskeleton reformation.

Gene ontology analysis was carried out similarly for differentially expressed genes in the female PFC. In females, 559 were differentially expressed due to adolescent ethanol exposure (Supplementary Table S1). Of those, 537 genes were unique to females and served as the input for female-specific gene ontology over-representation analysis using ToppFun (Figure 8A). The top significant categories for molecular function included cytokine binding, histone demethylase activity, and RNA helicase activity. The top significant categories for biological process included negative regulation of calcium transmembrane transporter activity, apoptotic process involved in development, and hormone-mediated signaling pathway (Figure 8C).

While the cytokine binding category (GO: 0005201) was found in both the male and female gene ontology analysis, there was a slight increase in the number of genes altered in females as compared to males. In males, six in this category were differentially expressed, while in females, the same six genes, and an additional five more were differentially expressed in this category. Similarly, both male and female gene ontology analysis returned the RNA helicase activity binding category (GO: 0030215), but with different genes represented. These results imply that the cytokine binding and RNA helicase activity are altered by adolescent ethanol exposure in both males and females, but may be altered through a different mechanism in each sex.

422 genes were differentially expressed in males, and 559 genes were differentially expressed in females due to adolescent ethanol exposure. Of those, only 22 genes were differentially expressed in both males and females (Figure 8A), and only 9 of 22 genes were annotated for use in our gene ontology analysis (*Ptgs1, Dgkg, Gatsl2, Sh3bp4, Smim3, Ado, Mc4r, Plau, Lman2l*). Due to the low number of annotated input genes, gene ontology analysis was not run on the genes differentially expressed due to ethanol in both male and female PFC.

#### 3.6.3 PFC differentially expressed genes due to the interaction of adolescent ethanol exposure and housing

In males, the interaction of adolescent ethanol exposure and single housing differentially regulated 931 total genes in the PFC (Supplementary Table S1). Of those, 842 genes were unique to males (Figure 9A) and served as the input for male-specific gene ontology over-representation analysis using ToppFun. Results of the gene ontology analysis are shown in Figure 9B. The top significant categories for molecular function included protein-hormone receptor activity, GTPase inhibitor activity, and complement binding. The top significant categories for biological process included regulation of protein ubiquitination and positive regulation of cytokine-mediated signaling pathway.

In females, 1989 genes were differentially expressed due to the interaction of adolescent ethanol exposure and single housing (Supplementary Table S1). Of those, 1900 genes were unique to females and served as the input for female-specific gene ontology over-representation analysis using ToppFun (Figure 9A). The top significant categories for molecular function included structural constituent of cytoskeleton, unfolded protein response, and DNA methyltransferase activity. The top significant categories for biological process included synaptic transmission – dopaminergic and regulation of cell projection assembly (Figure 9C).

931 genes were differentially expressed in males, and 1989 genes were differentially expressed in females due to the interaction of ethanol exposure and single housing throughout adolescence. Of those, 89 genes were differentially expressed in both males and females and were used as input into our gene ontology analysis (Figure 9A). Both males and females showed that the interaction of ethanol exposure and single housing during adolescence led to differential expression of genes related to cytokine binding (molecular function), calmodulin binding (molecular function), and cell–cell recognition (biological process) (Figure 9D).

### 3.7 Differentially expressed genes in the NAc

#### 3.7.1 NAc differentially expressed genes due to housing

In males, social isolation throughout adolescence induced differential regulation of 503 total genes in the NAc (Supplementary Table S2). Of those, 486 genes were unique to males (Figure 10A) and served as the input for male-specific gene ontology over-representation analysis using ToppFun. Results of the gene ontology analysis are shown in Figure 10B. The top significant categories for molecular function included active transmembrane transporter activity and transcription co-repressor binding. The top significant categories for biological process included regulation of action potential and regulation of astrocyte differentiation.

In females, 725 genes were differentially expressed in the NAc due to social isolation (Supplementary Table S2). Of those, 708 genes were unique to females and served as the input for female-specific gene ontology over-representation analysis using ToppFun (Figure 10A). The top significant categories for molecular function included G protein coupled glutamate receptor binding and calmodulin binding. The top significant categories for biological process included axon guidance, synapse assembly, and regulation of synapse organization (Figure 10C).

503 genes were differentially expressed in males, and 725 genes were differentially expressed in females due social isolation. Of those, only 17 genes were differentially expressed in both males and females (Figure 10A). Due to the low number of input genes, gene ontology analysis was not run on the genes differentially expressed due to single housing in both male and female NAc.

#### 3.7.2 NAc differentially expressed genes due to adolescent ethanol exposure

In males, adolescent ethanol exposure induced differential regulation of 859 total genes in the NAc (Supplementary Table S2). Of those, 806 genes were unique to males (Figure 11A) and served as the input for male-specific gene ontology over-representation analysis using ToppFun. Results of the gene ontology analysis are shown in Figure 11B. The top significant categories for molecular function included protein coupled receptor binding, calmodulin binding, and actin binding. The top significant categories for biological process included neurotransmitter transport, regulation of neuronal synaptic plasticity, learning, and dendrite development.

In females, 640 genes were differentially expressed in the NAc due to adolescent ethanol exposure (Supplementary Table S2). Of those, 587 genes were unique to females and served as the input for female-specific gene ontology over-representation analysis using ToppFun (Figure 11A). The top significant categories for molecular function included NADH binding, semaphorin receptor binding, and hormone binding. The top significant categories for biological process included synapse assembly, cellular response to calcium ion, and dendritic cell cytokine production (Figure 11C).

In the NAc, 859 genes were differentially expressed in males and 640 genes were differentially expressed in females due to adolescent ethanol exposure. Of those, 53 genes were differentially expressed in both males and females and were used as input into our gene ontology analysis (Figure 11A). Both males and females showed that adolescent exposure to ethanol led to differential expression of genes related to calcium channel activity (molecular function) and negative regulation of kinase activity (biological process) (Figure 11D).

#### 3.7.3 NAc differentially expressed genes due to the interaction of adolescent ethanol exposure and housing

In males, the interaction of adolescent ethanol exposure and single housing differentially regulated 803 total genes in the NAc (Supplementary Table S2). Of those, 731 genes were unique to males (Figure 12A) and served as the input for male-specific gene ontology over-representation analysis using ToppFun. Results of the gene ontology analysis are shown in Figure 12B. The top significant categories for molecular function cytokine activity and antioxidant activity. The top significant categories for biological process included positive regulation of action potential and glutathione metabolic process.

In females, 1939 genes were differentially expressed due to the interaction of adolescent ethanol exposure and single housing in the NAc (Supplementary Table S2). Of those, 1867 genes were unique to females and served as the input for female-specific gene ontology over-representation analysis using ToppFun (Figure 12A). The top significant categories for molecular function included calcium dependent protein binding, D3 dopamine receptor activity, synaptic receptor adaptor binding, and structural constituent of cytoskeleton. The top significant categories for biological process included cognition, vesicle mediated transport in synapse, and learning or memory (Figure 12C).

731 genes were differentially expressed in males, and 1939 genes were differentially expressed in females due to the interaction of ethanol exposure and single housing throughout adolescence. Of those, 72 genes were differentially expressed in both males and females and were used as input into our gene ontology analysis (Figure 12A). Both males and females showed that the interaction of ethanol exposure and single housing during adolescence led to differential expression of genes related to G protein-coupled peptide receptor binding (molecular function) and regulation of synapse assembly (biological process) (Figure 12D).

### 3.8 qPCR expression of immediate early genes in the PFC

To confirm some of our microarray findings, we tested a few immediate early genes that were significantly impacted by adolescent social isolation in the PFC. We assessed expression of *Arc*, *Erg1*, *Erg3*, and *Npas4* using qPCR on the same PFC tissue used for our microarray analysis (PND 57, *n* = 4/group). In our microarray data, these genes were significantly changed in the PFC due to housing or the interaction of housing and ethanol treatment (Supplementary Tables S1–S2).

**Figure 13:**
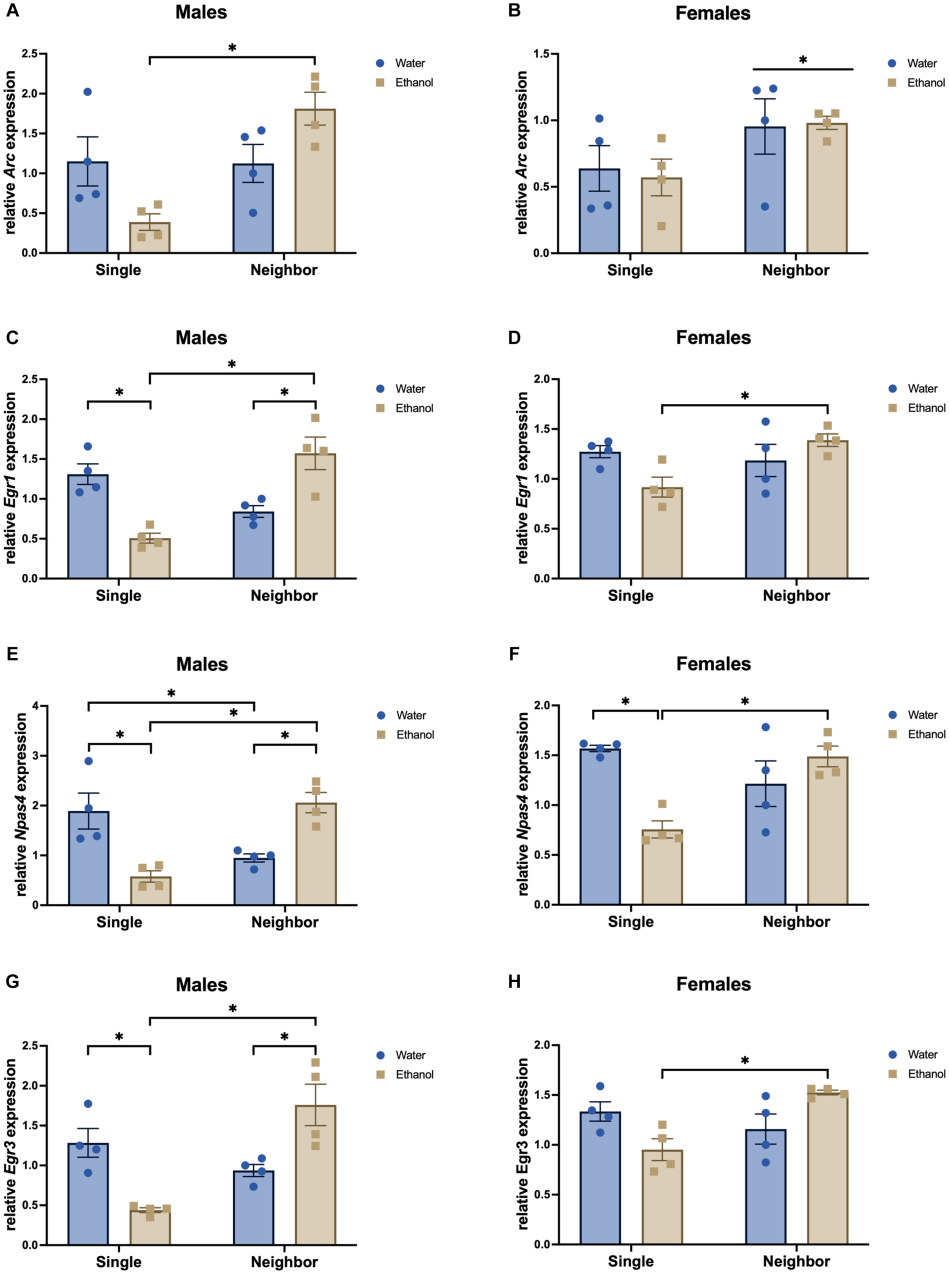
Immediate-early genes are changed in the PFC due to housing or the interaction of housing and ethanol treatment in males and females. Male and female DBA/2J mice were housed in same-sex groups in one of two conditions from post-natal day 29 (PND 29) until the end of the experiment: neighbor housed (4/complex) or single housed (1/cage). Single and neighbor-housed animals were given intermittent access to two bottles of water or a two-bottle choice between ethanol and water. qPCR was carried out on PFC tissue collected on PND 57 (*n* = 4/group) for the following genes: Arc, (A, B); Egr1 (C, D); Npas4 (E, F); Egr3 (G, H);. *p < 0.05 by two-way ANOVA.

In males, *Arc* (*F*_housing_ = 9.50, *p* = 0.009), *Egr1* (*F*_housing_ = 5.25, *p* = 0.041), and *Egr3* (*F*_housing_ = 8.93, *p* = 0.011), showed a significant decrease due to housing (Figures 13A,C,G), while *Npas4* was not altered by housing (*F*_housing_ = 1.52, *p* = 0.241, Figure 13E). No main effects of ethanol drinking were found (*Arc*: *F*_drinking_ = 0.027, *p* = 0.872; *Egr1*: *F*_drinking_ = 0.077, *p* = 0.786; *Npas4*: *F*_drinking_ = 0.214, *p* = 0.651; *Egr3*: *F*_drinking_ = 0.004, *p* = 0.950). All the genes we assessed showed a significant interaction of housing and ethanol drinking: *Arc* (*F*_interaction_ = 26.09, *p* = 0.008; single ethanol < neighbor ethanol), *Egr1* (*F*_interaction_ = 30.74, *p* < 0.0001; single water > single ethanol; neighbor water < neighbor ethanol; single ethanol < neighbor ethanol), *Npas4* (*F*_interaction_ = 30.67, *p* = 0.0001; single water > single ethanol, neighbor water < neighbor ethanol; single water > neighbor water; single ethanol < neighbor ethanol), *Egr3* (*F*_interaction_ = 26.09, *p* = 0.0003; single water > single ethanol, neighbor water < neighbor ethanol; single ethanol < neighbor ethanol).

In females, only *Arc* showed a significant decrease due to housing condition (*F*_housing_ = 5.60, *p* = 0.036, Figure 13B). Expression of *Egr1* (*F*_housing_ = 3.35, *p* = 0.092, Figure 13D), *Npas4* (*F*_housing_ = 4.071, *p* = 0.067, Figure 13F), and *Egr3* (*F*_housing_ = 3.52, *p* = 0.085 Figure 13H) were not significantly affected by housing condition. *Npas4* showed a trend for main effect of treatment (*F*_drinking_ = 4.07, *p* = 0.067). None of the other genes were significantly altered by adolescent drinking (*Arc*: *F*_drinking_ = 0.018, *p* = 0.896; *Egr1*: *F*_drinking_ = 0.527, *p* = 0.482; *Egr3*: *F*_drinking_ = 0.006, *p* = 0.940). With the exception of *Arc* (*F*_interaction_=, *p* = 0), all genes we assessed showed a significant interaction of housing and ethanol treatment: *Egr1* (*F*_interaction_ = 7.17, *p* = 0.02, single ethanol < neighbor ethanol), *Npas4* (*F*_interaction_ = 16.5, *p* = 0.002; single water > single ethanol, single ethanol < neighbor ethanol), *Egr3* (*F*_interaction_ = 12.58, *p* = 0.004; single ethanol < neighbor ethanol).

## 4 Discussion

Social isolation and ethanol use during adolescence can be detrimental to proper behavioral and cognitive development and show overlapping molecular and behavioral effects (Lodha and Brocato, 2022). The neighbor housing paradigm, where four standard mouse cages are fused together through four semi-permeable ports, allows for social interaction without direct contact (Pais et al., 2019). The goal of the current study was to determine whether the neighbor housing model could alleviate the behavioral and transcriptional changes in the PFC and NAc following social isolation, while still allowing for individual ethanol drinking. In our behavioral experiments, neighbor housing during early adolescence reduced anxiety-like behavior in the social interaction task and in the light–dark box as compared to single housed mice. It also rescued cognitive deficits in the novel object recognition task compared to single housed animals. Single housed males showed an increase in anxiety-like behavior compared to neighbor housed males, while single housed females showed a more moderate increase in their anxiety phenotypes as compared to neighbor housed females. Adolescent ethanol drinking did not alter mouse performance in the social, cognitive, or anxiety-like behavior tasks in either sex. Different housing paradigms did not alter 2-BC ethanol drinking behavior in adolescents or in adults. Prior ethanol drinking in a 2-BC paradigm also did not significantly alter adult drinking behavior.

The majority of the observed behavioral effects were due to housing condition and ethanol drinking did not further modulate adult social, cognitive or anxiety-like behaviors. Studies from our lab have previously shown that binge ethanol in DBA/2 J adolescents (4 g/kg by gavage) did not lead to lasting changes in social interaction or anxiety-like behavior supporting the data shown here (Bent et al., 2022). However, our findings are in contrast to an earlier report that binge ethanol in adolescent DBA/2J mice (4 g/kg by gavage) causes memory deficits in the novel object recognition task (Wolstenholme et al., 2017). This discrepancy is likely because mice in the current study did not consume as much ethanol during the voluntary, 2-BC paradigm as during the oral gavage paradigm. The differences in behavioral results from the current study and previous studies could also be due to the extended period of ethanol abstinence in the current study. Future experiments using the drinking in the dark model could increase peak blood ethanol concentrations and may better model binge drinking in adolescents. Anxiety-like behavior was decreased in neighbor housed mice relative to single housing, and stronger in males. This was not modified by a history of ethanol drinking. Adolescent social isolation studies do not always find effects on anxiety-like behavior and are frequently confounded by heightened locomotion in single housed animals (Lodha and Brocato, 2022). We also observed differences in total locomotion in the light–dark model, where neighbor mice were more locomotive than single housed mice, which could suggest that neighbor housing may alter other unmeasured aspects of anxiety-like behavior. A history of ethanol drinking did not alter social or anxiety-like behavior in this study and this is consistent with other findings in the field. Negative affective states following ethanol withdrawal or during protracted abstinence are not always found and may depend on strain, assay and length of abstinence (Bloch et al., 2022; Lodha and Brocato, 2022).

Somewhat surprisingly, housing condition did not alter ethanol intake or preference in the 2-BC model. Drinking in proximity to another mouse, but not in the same physical space, did not affect ethanol intake in males or females. Social isolation tends to lead to increased ethanol intake in adolescent rats (Schenk et al., 1990; Wolffgramm, 1990; Chappell et al., 2013; Skelly et al., 2015) and mice (Advani et al., 2007; Lopez et al., 2011; Sanna et al., 2011) as compared to group housing. A few studies have used a semi-permeable partition cages in adult rats and mice (Wolffgramm, 1990; Tomie et al., 2015), but ethanol intake did not always differ between group housed and single housed rodents (Palm and Nylander, 2014). Females seem to be more sensitive to the social stimulating effects and will drink increasing amounts of ethanol in the presence of increasing number of cage mates; this effect was absent in males (Tomie et al., 2015). Social experience without physical contact may be a stress-inducing experience, which could lead to increased ethanol intake. For example, living under a partition condition worsened recovery from surgery in female mice and increased physiological measures of heart rate, suggesting that living under partition housing was more stressful than single or group housing (Van Loo et al., 2007). It is likely that neighbor housing could not fully ameliorate all the issues associated with even semi-social isolation and the normal trajectory of brain development is still altered by having only olfactory and visual contact with conspecifics. Indeed, our bioinformatic analyses suggests differential pathways are activated by these housing conditions. However, these studies have not included a more typical group housed condition as a comparison group to determine signaling pathways specifically altered by housing in the neighbor cages in adolescence.

Adolescent drinking did not increase adult 2-BC intake or preference in either the neighbor or single housed mice. We did observe a temporary increase in ethanol intake in single housed males without a history ethanol in comparison to single housed mice with a history of ethanol. Adolescent social isolation tends to increase ethanol drinking in adulthood as compared to their group housed counterparts (Advani et al., 2007; McCool and Chappell, 2009; Chappell et al., 2013; Butler et al., 2014; Lesscher et al., 2015; Lopez and Laber, 2015; Skelly et al., 2015). The present results did not indicate a difference in adult ethanol drinking between single and neighbor housed mice. This could be because the mice were already consuming ethanol close to their maximal capacity and thus, are experiencing a ceiling effect. Alternatively, being subjected to single or neighbor housing conditions since adolescence could have produced a stronger adverse effect that elevated adult drinking in all groups. This could have precluded our ability to see effects solely from adolescent ethanol history.

Our bioinformatics analysis revealed sets of common themes altered by housing or drinking that may affect the trajectory of PFC development. In the current study, social isolation and drinking occurred during adolescence when synaptic pruning, increased myelination and proper excitatory/inhibitory balance in the PFC is necessary for proper adult development and behavioral responses (Spear, 2013; Lodha and Brocato, 2022; Tetteh-Quarshie and Risher, 2023). Social isolation and/or binge drinking in adolescence can disrupt these processes (Lodha and Brocato, 2022) and our differential gene analysis has identified altered gene expression in related categories. For example, many gene ontology categories related to the extracellular matrix or cytoskeleton were impacted due to housing or ethanol consumption: extracellular matrix binding (in male PFC due to housing), cell projection assembly (in female PFC due to interaction of housing and ethanol), structural constituent of cytoskeleton (in female PFC due to interaction of housing and ethanol), and actin cytoskeleton reformation (in male PFC due to ethanol). During adolescent brain development, the extracellular matrix supports processes involved in plasticity (Gundelfinger et al., 2010). Thus, alterations in the structure and function of the extracellular matrix could lead to lasting changes in the brain and have been suggested to underlie neuropsychiatric disease (Lubbers et al., 2014). We also saw a number of inflammation categories in the PFC analyses. These included complement binding (in male PFC due to interaction of housing and ethanol), cytokine binding (in male and female PFC due to interaction of housing and ethanol, in male PFC due to ethanol, in female PFC due to ethanol), cytokine-mediated signaling pathway (in male PFC due to housing), and cell–cell recognition (in male and female PFC due to interaction of housing and ethanol). The impact of adolescent binge ethanol on inflammation pathways has been well-studied by other groups (Pascual et al., 2018; Doremus-Fitzwater and Deak, 2022; Crews et al., 2023; Nwachukwu et al., 2023), and this finding helps to support those data.

In the PFC, both single housing and drinking in adolescence altered genes related to epigenetic regulation of gene transcription, specifically histone demethylase activity (in female PFC due to drinking) RNA helicase activity (in male and female PFC due to drinking), methyl CpG binding (in female PFC due to housing), and DNA methyltransferase activity (in female PFC due to an interaction between housing and drinking). Similar over-represented gene ontology categories were also found to be differentially regulated following binge ethanol in the PFC of adolescent DBA/2J mice in prior studies (Wolstenholme et al., 2017; Brocato and Wolstenholme, 2023). Epigenetic regulation through histone demethylase activity is particularly intriguing as a growing number of studies show ethanol alters methylation marks (Bohnsack and Pandey, 2021; Brocato and Wolstenholme, 2021; Siomek-Gorecka et al., 2021; Jarczak et al., 2023) and inhibits the production of methyl substrates (Watson et al., 2011; Brocato and Wolstenholme, 2021; Jarczak et al., 2023).

Within the NAc, categories related to synaptic plasticity were over-represented, largely altered by ethanol drinking. For example, genes related to synapse assembly (in male and female NAc due to interaction of housing and ethanol, in female NAc due to ethanol), synapse organization (in female NAc due to housing), neuronal synaptic plasticity (in male NAc due to ethanol), dendrite development (in male NAc due to ethanol), and learning (in female NAc due to interaction of housing and ethanol, in male NAc due to ethanol) were observed.

Several gene ontology categories found in the current study aligned with results of other studies that have suggested GABAergic (in male PFC due to housing), serotonergic (in female PFC due to housing), and dopaminergic neurotransmission (in female PFC due to interaction of housing and ethanol) are altered by social isolation (Bibancos et al., 2007; Pisu et al., 2011; Talani et al., 2016; Lander et al., 2017). It is possible that behavior is being influenced through different mechanisms in the PFC and the NAc. In the PFC, our results indicated changes to genes related to the excitatory/inhibitory balance, while in the NAc, our results indicated changes to genes related to synaptic structure and function. Interestingly, housing and ethanol appear to regulate similar processes in females as both methylase activity and apoptotic signaling were over-represented in the female PFC analyses.

Reduced sociability, i.e., social preference, social recognition, and social exploration, and increased anxiety-like behaviors are frequently observed in socially isolated animals as compared to their group-housed counterparts (Lodha and Brocato, 2022). A lack of social experience during adolescence could reduce the necessary stimulation from brain regions involved in social behavior such as the PFC and NAc, potentially giving rise to impaired development of neural connections and produce lasting changes in behavior. In our qPCR follow-up study, we examined a set of immediate early genes, *Arc*, *Npas4*, *Egr1*, and *Egr3*, that are regulated in an activity-dependent manner to alter activity- and plasticity-associated gene expression. *Arc* is an immediate early transcription factor and is epigenetically reduced in adolescent drinking mice (Kyzar et al., 2019). It plays a role in increased adult drinking following adolescent ethanol exposure and may mediate withdrawal-induced anxiety-like behaviors (Kyzar et al., 2019; Bohnsack et al., 2022). *Npas4* is an immediate early gene (IEG) activated by synaptic activity and regulates inhibitory synapse development (Lin et al., 2008). *Egr1* is an IEG induced by acute stress or ethanol, but downregulated in models of social stress and chronic ethanol exposure (Duclot and Kabbaj, 2017). It is a major mediator and regulator of synaptic plasticity and neuronal activity and plays a role in memory consolidation and can activate *Arc* (Duclot and Kabbaj, 2017). *Egr3* also plays a significant role in learning and memory (Li et al., 2007). Surprisingly, despite our microarray ANOVA results showing a positive main effect of housing, these IEGs were not significantly altered by housing condition in 2-way ANOVA analysis of the qPCR data, which could suggest that an experience-dependent activation may first be needed to detect alterations in IEG signaling. However, neighbor housing did alter the response to ethanol for most of these genes in both sexes. With the exception of *Arc* in female PFC, each of these genes showed a significant interaction with ethanol drinking. A history of ethanol drinking appears to downregulate expression of IEGs in single housed mice (male *Egr1*, *Npas4*, *Egr3*, female *Npas4*) suggesting that chronic ethanol drinking blunts immediate early gene expression. However, the opposite effect occurs in neighbor housed mice – expression of *Egr1*, *Npas4*, and *Egr3* in males (with a similar nonsignificant trend in females) was increased in ethanol drinking mice. It is possible that neighbor housing provides social stimulation and environmental enrichment, allowing for ethanol exposure to induce the activation of immediate early genes and downstream signaling cascades. However, single housing reduces social stimulation and environmental enrichment and, thus, expression of these genes is decreased in the presence of ethanol. Prior studies have shown that ethanol can blunt context-dependent responses in many of the IEGs explored here. Ethanol exposure during the prenatal period decreased expression of *Arc*, *Egr1*, *Npas4*, and c-*Fos* (Heroux et al., 2019) during a context exposure paradigm. Intermittent ethanol exposure during adolescence (5 g/kg by gavage) blunted *cFos* and *Egr1* expression in the PFC of rats following an ethanol challenge in adulthood (Liu and Crews, 2015). In our behaviorally naïve mice, these activity-dependent immediate early genes were reduced by ethanol drinking, but only in the single housed mice. These data suggest that social isolation could be disrupting the basal level of activity in the PFC as compared to neighbor housing and this differential level of PFC activity leads to opposite regulation of these immediate early genes in the neighbor housed mice following ethanol drinking.

Taken together, social stress experienced in adolescence influences social and cognitive behaviors. The direction of change was similar in both sexes, where increased exposure to social isolation stress during adolescence decreased sociability and cognitive function and increased anxiety-like behavior in the light–dark box. These behavioral changes were not significantly influenced by adolescent ethanol consumption. Surprisingly, while the neighbor environments altered cognitive and affective behaviors, drinking behavior did not differ between housing conditions. Prior exposure to ethanol in adolescence also did not impact adult ethanol drinking behavior in a 2-BC model. Thus, these studies have shown the importance of appropriate peer to peer social interaction during adolescence for proper development of brain circuitry and age-appropriate behavioral outcomes. Housing conditions that allow for partial contact between conspecifics such as the neighbor housing model may be able to ameliorate some of the behavioral and neurobiological changes induced by social isolation, but not within all behavioral domains. These studies have begun to describe some of the brain cell signaling alterations following social isolation and or ethanol drinking. Many of the overrepresented pathways were in common with other prior studies showing that social isolation and drinking in adolescence may converge upon the same signaling pathways, but that the degree of overlap is not fully understood. Importantly, our data on immediate early gene expression documents a dramatic change induced by neighbor housing on the response of these genes to chronic ethanol consumption. This may have implications at the level of neural circuit activation in single versus neighbor housed animals during ethanol consumption.

## Data availability statement

The datasets generated for this study can be found in the GEO database (Accession # GSE242910; https://www.ncbi.nlm.nih.gov/geo/query/acc.cgi).

## Ethics statement

The animal study was approved by VCU Institutional Animal Care & Use Committee. The study was conducted in accordance with the local legislation and institutional requirements.

## Author contributions

JL: Formal analysis, Investigation, Validation, Writing – review & editing. EB: Formal analysis, Investigation, Methodology, Validation, Visualization, Writing – original draft, Writing – review & editing. MN: Data curation, Formal analysis, Visualization, Writing – original draft. MMM: Investigation, Writing – review & editing. ACP: Investigation, Methodology, Validation, Writing – review & editing. ABP: Investigation, Methodology, Writing – review & editing. MFM: Resources, Writing – review & editing. JW: Conceptualization, Formal analysis, Funding acquisition, Methodology, Resources, Supervision, Validation, Visualization, Writing – original draft, Writing – review & editing.
