# Supplementary material for: Adolescent social housing protects against adult emotional and cognitive deficits and alters the PFC and NAc transcriptome in male and female C57BL/6J mice
Source: Front Neurosci. 2023 Dec 7;17:1287584. doi: 10.3389/fnins.2023.1287584 (PMC10733512; doi:10.3389/fnins.2023.1287584)
Supplement: Supplementary file 1 [file Image_1.pdf]

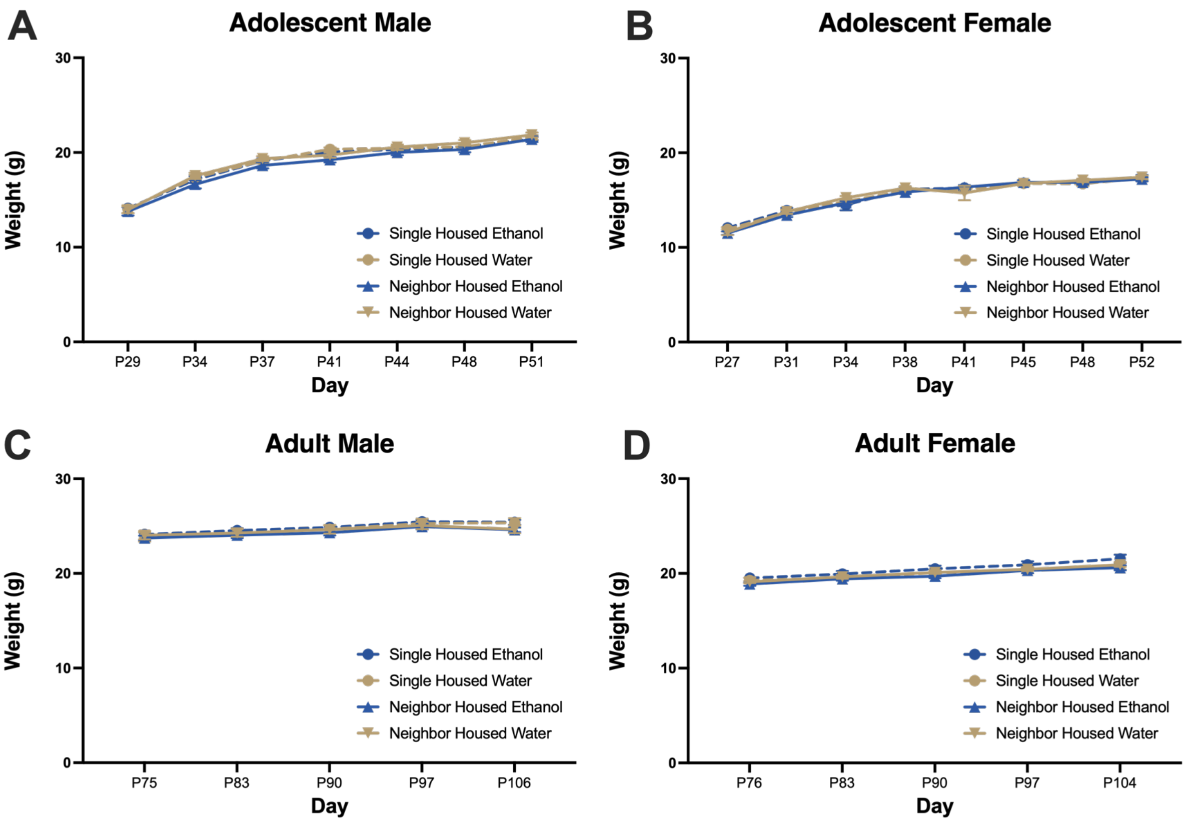

Supplementary Figure S1: Body weight is not altered by housing or ethanol access. Body weight of adolescent males (A), adolescent females (B), adult males (C) and adult females (D).
